# Whole transcriptomic analysis of the plant-beneficial rhizobacterium *Bacillus amyloliquefaciens* SQR9 during enhanced biofilm formation regulated by maize root exudates

**DOI:** 10.1186/s12864-015-1825-5

**Published:** 2015-09-07

**Authors:** Nan Zhang, Dongqing Yang, Dandan Wang, Youzhi Miao, Jiahui Shao, Xuan Zhou, Zhihui Xu, Qing Li, Haichao Feng, Shuqing Li, Qirong Shen, Ruifu Zhang

**Affiliations:** Jiangsu Key Lab for Organic Solid Waste Utilization, National Engineering Research Center for Organic-based Fertilizers, Jiangsu Collaborative Innovation Center for Solid Organic Waste Resource Utilization, Nanjing Agricultural University, 210095 Nanjing, China; Key Laboratory of Microbial Resources Collection and Preservation, Ministry of Agriculture, Institute of Agricultural Resources and Regional Planning, Chinese Academy of Agricultural Sciences, 100081 Beijing, China; College of Resources & Environmental Science, Nanjing Agricultural University, 210095 Nanjing, China

**Keywords:** Plant growth-promoting rhizobacteria, Genome, Root exudates, Biofilm, Transcriptome

## Abstract

**Background:**

*Bacillus amyloliquefaciens* SQR9 is a plant growth-promoting rhizobacteria (PGPR) with outstanding abilities to enhance plant growth and to control soil-borne diseases. Root exudates is known to play important roles in plant-microbe interactions. To explore the rhizosphere interactions and plant-beneficial characteristics of SQR9, the complete genome sequence as well as the transcriptome in response to maize root exudates under biofilm-forming conditions were elucidated.

**Results:**

Maize root exudates stimulated SQR9 biofilm formation in liquid culture, which is known to be positively correlated with enhanced root colonization. Transcriptional profiling via RNA-sequencing of SQR9 under static conditions indicated that, at 24 h post-inoculation, root exudates stimulated the expression of metabolism-relevant genes, while at 48 h post-inoculation, genes related to extracellular matrix production (*tapA-sipW-tasA* operon) were activated by root exudates. The individual components in maize root exudates that stimulated biofilm formation included glucose, citric acid, and fumaric acid, which either promoted the growth of SQR9 cells or activated extracellular matrix production. In addition, numerous groups of genes involved in rhizosphere adaptation and in plant-beneficial traits, including plant polysaccharide utilization, cell motility and chemotaxis, secondary antibiotics synthesis clusters, and plant growth promotion-relevant, were identified in the SQR9 genome. These genes also appeared to be induced by the maize root exudates.

**Conclusions:**

Enhanced biofilm formation of *B. amyloliquefaciens* SQR9 by maize root exudates could mainly be attributed to promoting cell growth and to inducing extracellular matrix production. The genomic analysis also highlighted the elements involved in the strain’s potential as a PGPR. This study provides useful information for understanding plant-rhizobacteria interactions and hence for promoting the agricultural applications of this strain.

**Electronic supplementary material:**

The online version of this article (doi:10.1186/s12864-015-1825-5) contains supplementary material, which is available to authorized users.

## Background

Plant growth-promoting rhizobacteria (PGPR) are a group of rhizosphere-colonizing bacteria that can promote plant growth and control soil-borne diseases, which are of great importance in both basic and applied microbiology [1, 2]. *Bacillus spp.* are important members of the PGPR, and have been commercially exploited as biofertilizers and biocontrol agents [3, 4]. Complete genome sequencing of several plant-associated *Bacillus amyloliquefaciens* strains, including FZB42, CAU B946, and YAU B9601-Y2, have revealed functional genes involved in growth promotion (genes related to the synthesis of plant hormones) and disease control (gene clusters involved in nonribosomal synthesis of lipopeptides and polyketides with antibiotic activity) [4–7].

Detailed investigations of the interactions between plants and root-associated PGPR have been performed to determine the requirements for the bacteria to adapt and colonize roots, providing useful information regarding the potential applications of the PGPR strains in agriculture [8, 9]. Increasing evidence supports the notion that plant-bacteria interactions mainly occur in the rhizosphere and are regulated by root exudates [10–14]. For instance, flavonoids secreted by roots of leguminous plants are known to play an important role in the early signaling events of legume-rhizobia interactions [12]; while organic acids, such as malic acid and citric acid, in root exudates recruit *Bacillus* strains in the rhizosphere [15, 16].

High-throughput strategies, including microarray analyses [8, 9, 17, 18], cDNA-based suppression-subtractive hybridization [19], and promoter trapping [20], have been applied to investigate bacterial interactions with root exudates (*in vitro*) and with the root surface (*in vivo*). The groups of genes involved in plant-microbe interactions consist mainly of (families of) genes involved in metabolism, bacterial motility and chemotaxis, transport, secretion, and antibiotics production [8, 9, 17, 18, 21]. Next-generation sequencing (NGS) technologies have provided new opportunities to perform whole-genome sequencing and to investigate dynamic transcriptomes [22]. In addition, NGS methods for RNA analysis (including RNA-Seq) have been used in studies of small regulatory RNAs [23] and genome annotation [24]. These technologies have been especially useful in measuring the transcript expression levels under different conditions [25, 26], both for eukaryotes [27, 28] and prokaryotes [24]. Such high-throughput techniques are time-saving and useful in investigating the uncharacterized genes. Compared with conventional strategies, such as microarray analysis, RNA-Seq offers a better way to study root exudates-bacterium interactions.

*B. amyloliquefaciens* SQR9 was isolated from the plant rhizosphere and is able to reduce attack by the phytopathogenic fungus *Fusarium oxysporum* f. sp. *cucumerinum* J. H. Owen (FOC) through efficient root colonization followed by production of antifungal metabolites [29–32]. SQR9 colonizes roots more efficiently and promotes plant growth better than other root-associated *Bacillus* strains [33]. Products derived from SQR9 are also widely used in agriculture in China under the BIO™ trademark. Therefore, *B. amyloqiquefaciens* SQR9 could be regarded as an ideal PGPR strain for exploring rhizoshphere plant-microbe interactions.

Biofilm formation has been found to be crucial to colonization and expression of beneficial traits by PGPR strains [34, 35]. Importantly, it was found that the *in vitro* addition of maize root exudates could stimulate biofilm formation of SQR9. Although several studies have explored the plant-microbe interactions through different high-throughput approaches, few investigations have been performed under biofilm-formation condition. In this study, to further explore the mechanisms involved in enhanced biofilm formation of SQR9 as regulated by maize root exudates, as well as its regulatory roles on other PGP-relevant functional genes, the complete genome sequence of SQR9 was determined by Roche 454 pyrosequencing to provide a the reference map for transcriptomic analysis. Then, the transcriptional profiling was investigated by Illumina RNA-Seq. The results of this study reveal the genetic basis of rhizosphere adaption and plant beneficial effects of SQR9, which are crucial for understanding plant-rhizobacteria interactions and improving the application of this strain in agriculture.

## Results

### Plant-beneficial activities of *Bacillus amyloliquefaciens* SQR9

Greenhouse experiments were performed to evaluate the effects of *B. amyloliquefaciens* SQR9 on the growth of maize, one of the most important and widely-planted grain crops in the world. To avoid the confounding influence of mycoprotein on seedlings, equal amounts of inactivated SQR9 cells were used as a control. The results revealed that SQR9 significantly promoted the growth of maize plants. When live bacterial suspensions were applied, maize biomass, shoot height, root length, and root surface area were significantly greater than the control by 42–60 %, 32–46 %, 33–49 %, and 29–59 %, respectively (Table 1). This outstanding plant-promoting performance indicates that SQR9 can be regarded as an ideal PGPR agent.

**Table 1:** Effect of *Bacillus amyloliquefaciens* SQR9 on the growth of maize seedlings

Treatment	Dry weight (g)	Height (cm)	Root length (cm)	Root surface area (cm^2^)
CK1	3.59 ± 0.45c	50.81 ± 5.61c	1335.87 ± 26.65c	354.74 ± 14.58c
CK2	3.86 ± 0.23c	51.84 ± 3.64c	1351.42 ± 26.89c	355.55 ± 11.57c
T1	5.08 ± 0.62b	67.08 ± 2.67b	1775.04 ± 20.79b	458.47 ± 13.76b
T2	6.16 ± 0.45a	75.81 ± 3.43a	2016.47 ± 54.66a	564.27 ± 15.63a

Different letters indicate significant differences at *P* < 0.05 using Duncan’s multiple range tests. CK1: seedlings inoculated with suspensions of 5 mL inactivated bacteria (10^8^ CFU · mL^-1^); CK2: seedlings inoculated with suspensions of 10 mL inactivated bacteria (10^8^ CFU · mL^-1^); T1: seedlings inoculated with suspensions of 5 mL bacteria (10^8^ CFU · mL^-1^); T2: seedlings inoculated with suspensions of 10 mL bacteria (10^8^ CFU · mL^-1^). Plants were grown in a greenhouse for 55 days (*n* = 10)

### Interaction of maize and *B. amyloliquefaciens* SQR9: root colonization and stimulation of biofilm formation by root exudates

**Fig. 1:**
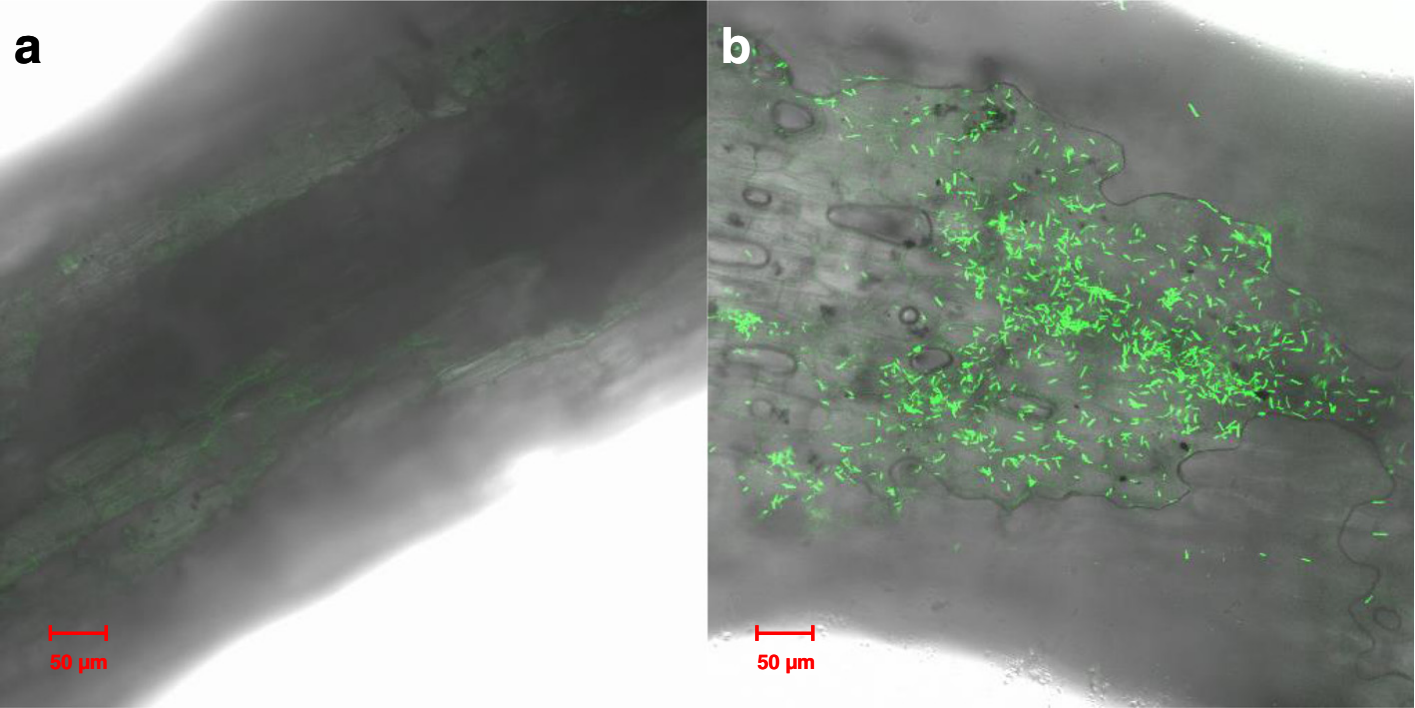
Colonization of maize roots by SQR9-*gfp* cells. Uninoculated control roots of maize seedlings (**a**) and roots inoculated with SQR9-*gfp* cells (**b**) were imaged by confocal laser scanning microscopy (CLSM) at 5-days post-inoculation

Understanding of the interaction mechanisms between host plants and PGPRs is important for practical application of these agents. Root colonization of inoculated PGPR agents is considered as a prerequisite for successful growth promotion and biocontrol activities [36]. Confocal laser scanning microcopy (CLSM) indicated that, after 5 days of incubation in a gnotobiotic soil system, the green fluorescence protein (GFP)-tagged SQR9 cells colonized the maize root very well and formed biofilms on the roots with a density of approximately 1.8 × 10^6^ CFU · g^-1^ root (Fig. 1).

**Fig. 2:**
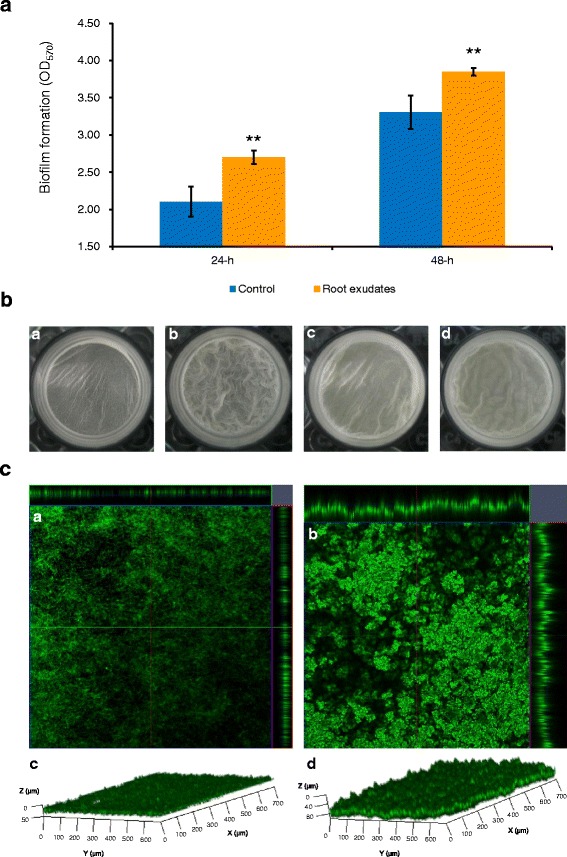
Effects of concentrated maize root exudates on biofilm formation of SQR9. **a** Effects of maize root exudates on the biomass of biofilm formed by SQR9. Data with asterisks were significantly different from the control at each time point (*, *P* < 0.05; **, *P* < 0.01; *t* test). **b** Effects of root exudates on the appearance of biofilm formed by SQR9: *a*, Control, 24 h post-inoculation; *b*, Treatment with maize exudates, 24 h post-inoculation; *c*, Control, 48 h post-inoculation; *d*, Treatment with maize exudates, 48 h post-inoculation). **c** Effects of root exudates on the three-dimensional structure of a biofilm formed by SQR9 visualized by confocal laser scanning microscopy (CLSM) 24 h post-inoculation. *a, c*. Control; *b, d*. Treatment with maize root exudates

Root colonization of *Bacillus* strains was indicated to be positively correlated with their ability to form biofilms under laboratory condition [31, 35, 37]. Since root exudates are important in rhizosphere dialogues and in biofilm formation on plant roots by PGPR strains [1], static culture assays were performed to evaluate the effects of maize root exudates of various concentrations on SQR9 biofilm formation. The addition of 1 × and 2 × maize root exudates in 1/2 MSgg medium significantly enhanced the biofilm formation of SQR9 compared with the control, as revealed by both increased biomass and more complex architecture observed using CLSM (Fig. 2; Additional file 1: Figure S1); these two treatments did not differ significantly from one another. The 0.5 × root exudates also stimulated biofilm formation not significantly so (Additional file 1: Figure S1).

### Experiments design for investigating the transcriptional profiling of SQR9 in response to maize root exudates during biofilm formation

To investigate the regulatory roles of maize root exudates on the rhizosphere behavior of SQR9, especially the mechanisms involved in the induced biofilm formation, a high-throughput Illumina RNA-Seq was performed to study the transcriptomic response of SQR9 to maize root exudates. Based on the results obtained above, a 1 × concentration of maize root exudates was used in this experiment. Considering the dynamics curves of biofilm formation, cells were collected and RNA extracted 24 and 48 h post-inoculation, which represented the mid-exponential phase (biomass quickly increasing) and stationary phase (biomass peaked and remained stable) during the biofilm formation, respectively (Additional file 1: Figure S1). Thus, two treatments (addition of 1× maize root exudates or the negative control) and two sampling points (24 and 48 h post-inoculation) for cell harvesting were used in the transcriptomic analysis (see Methods).

### Genomic analysis of *Bacillus amyloliquefaciens* SQR9

**Fig. 3:**
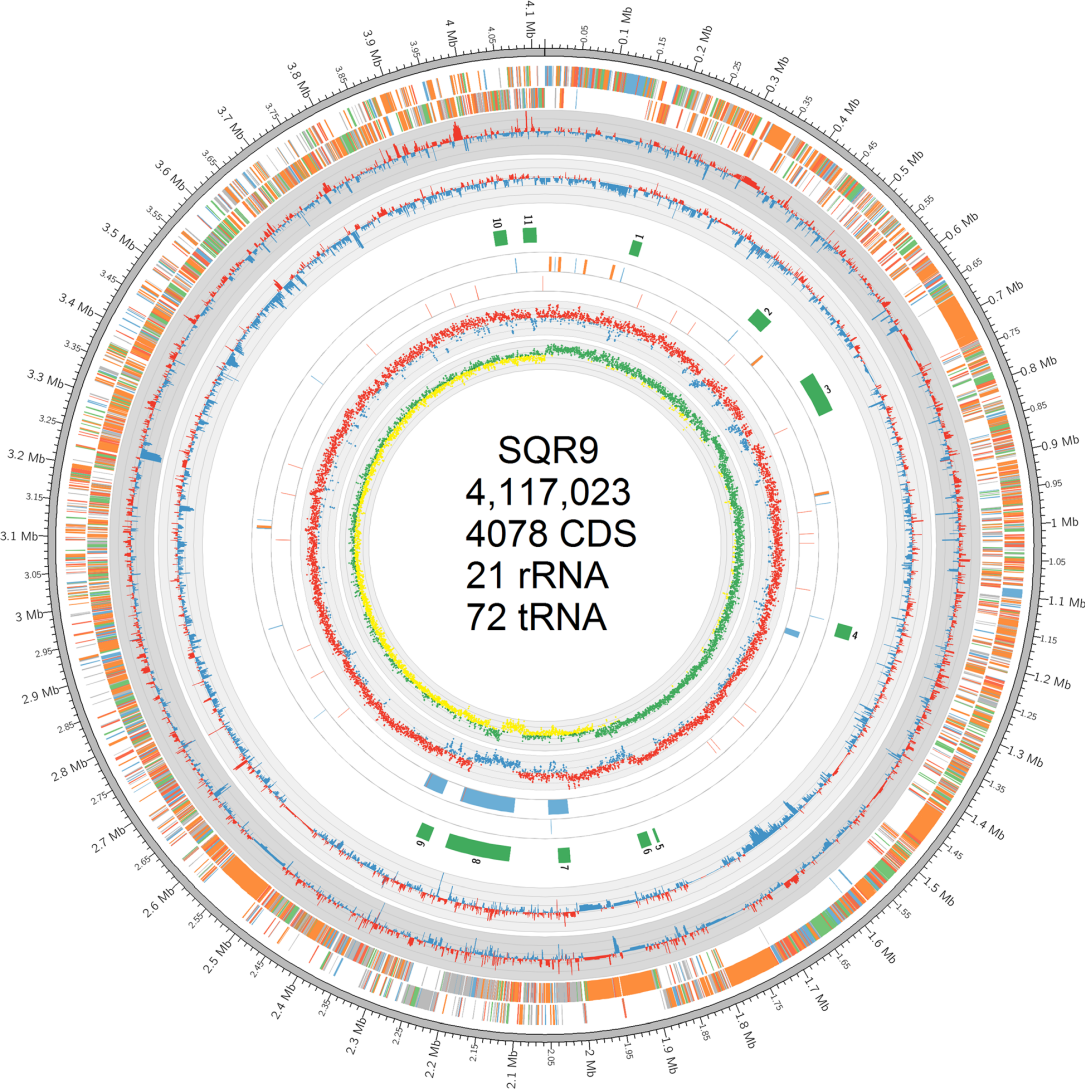
Circular map of the *Bacillus amyloliquefaciens* SQR9 genome. The gene expression profiles in response to maize root exudates are also shown. 1^st^ circle: all genes in color code according to function: orange, information storage and processing; green, cellular processes and signaling; purple, metabolism; red, poorly characterized, red; gray, unknown; 2^nd^ and 3^rd^ circle: gene expression responses to root exudates after inoculation for 24 h and 48 h, respectively; 4^th^ circle: the numbered 11 DNA islands (green); 5^th^ circle: rRNAs (orange) and tRNAs (blue); 6^th^ circle: prophages (green) and IS elements (red); 7^th^ circle: GC content; 8^th^ circle: GC skew. The highlighted area of gray is a conserved genomic island of SQR9, and the green area is a shared prophage with *Bacillus subtilis* 168

**Fig. 4:**
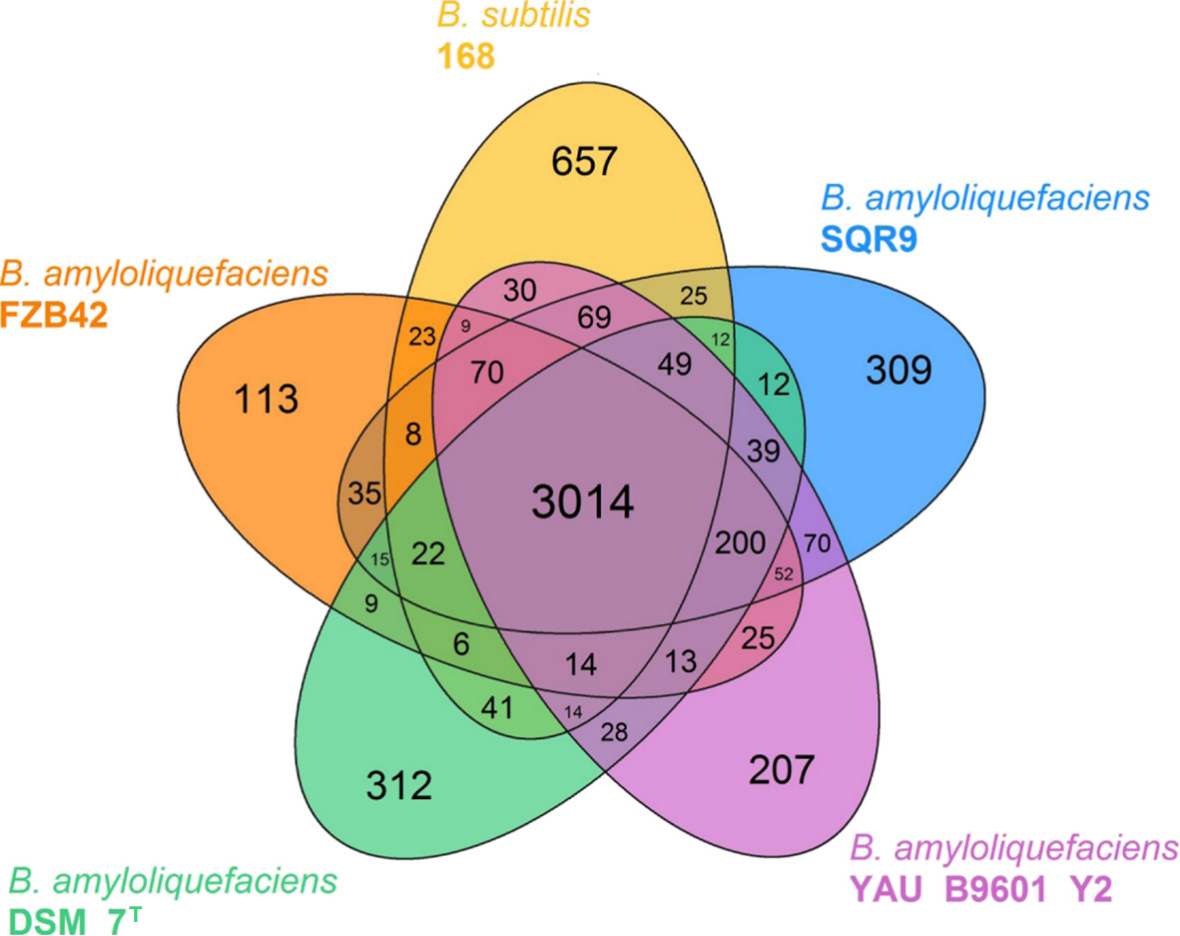
Venn diagram showing the genes encoded by four *Bacillus amyloliquefaciens* strains and *Bacillus subtilis* 168. The core genes are those located at the intersection of the five circles

To provide a mapping background for the transcriptomic analysis, whole-genome sequencing of SQR9 was performed using Roche 454 high-throughput pyrosequencing technology. The general features of the SQR9 genome and other relevant *Bacillus* strains are summarized in Table 2. The single circular chromosome (4,117,023-bp) with a GC content of 46.1 % encodes 4,078 predicted proteins, 72 tRNA genes, 7 rRNA operons, 218 prophages-associated genes, and 358 non-coding RNAs (Fig. 3; Additional file 2: Table S1). The core genomes of SQR9 and four other closely related *Bacillus* strains (*B. subtilis* strain 168 and *B. amyloliquefaciens* strains FZB42, DSM7^T^, and B9601-Y2) consists of 3,014 orthologous genes and a pan genome size of 5,643 orthologous genes, among which 309 genes were unique to SQR9 (Fig. 4; Additional file 3: Table S2). Pairwise genome and gene order comparisons suggested that the majority of the SQR9 protein-encoding sequences were conserved in 168 and FZB42 (Additional file 4: Figure S2). An ortholog analysis within the three strains revealed that 304 genes were shared by SQR9 and FZB42, while only 156 genes were shared by SQR9 and 168 (Fig. 4; Additional file 5: Table S3). A phylogenetic tree constructed from the core genomes of SQR9 and 17 additional *Bacillus* strains indicated that SQR9 belonged to the *B. amyloliquefaciens* group (Additional file 6: Figure S3).

**Table 2:** Genomic features of the *Bacillus amyloliquefaciens* SQR9 genome. The SQR9 genome was compared with those of *Bacillus subtilis* 168 and three other *B. amyloliquefaciens* strains

Features	*B. amyloliquefaciens* SQR9	FZB42	DSM7^T^	B9601-Y2	*B. subtilis* 168
Genome size (bp)	4,117,023	3,918,589	3,980,199	4,242,774	4,214,630
G + C content (mol %)	46.1	46.4	46.1	45.85	43.5
Protein-coding sequences	4078	3693	3921	3989	4106
Average CDS size (bp)	916	933	888	927	895
Percent of coding region	89 %	88 %	87 %	87 %	87.2 %
Ribosomal RNA operons	7	10	10	10	10
Number of tRNAs	72	89	94	91	86
Phage-associated genes	218	44	n.r.	n.r.	268
Transposase genes of IS elements	28	9	n.r.	24	0

n.r., not reported

Genomic islands (GI) prediction by IslandViewer and M-GCAT identified 11 large regions of genomic plasticity (Additional file 7: Table S4). Importantly, GI3 was a unique mobile genomic fragment that is not found in the genomes of other *Bacillus* strains. It consists of genes related to the biosynthesis of an unknown polyketide (Additional file 8: Figure S4).

### Overall transcriptional profiling of SQR9 in response to maize root exudates during the biofilm formation process

The RNA-Seq generated approximately 10 million reads for each sample, of which 60–70 % were confirmed to be valid after filtering reads with Phred quality scores of < 20 using FASTX-Toolkit version 0.0.13.2 (http://hannonlab.cshl.edu/fastx_toolkit/index.html). The rarefaction curves revealed that the sequence throughput was sufficient to cover the whole SQR9 genome (Additional file 9: Figure S5), suggesting that the data were adequate for transcriptomic profiling studies.

Based on the standards for identifying genes with significantly different expression levels between different treatments [expression fold-change ≥ 1.5, *q*-value ≤ 0.001 (false discovery rate, FDR), and a RPM (reads per million) consistently above 10 in at least one treatment], genes representing roughly 15.8–25.1 % of the SQR9 transcriptome were significantly regulated by the addition of root exudates as compared with control (Table 3; Additional file 10: Figure S6). Of the 643 significantly affected genes after the 24-h incubation, 443 were annotated with known functions; most belonged to the categories of metabolism and ATP-binding cassette (ABC) transporters (Table 4). The remaining 200 genes were annotated to encode putative enzymes, hypothetical proteins, and proteins with unknown function. Once the biofilm had matured, at 48 h post-treatment, most of the differentially expressed genes were down-regulated. Of the 1,024 differentially regulated genes, 758 with known functions were mainly assigned to functions related to metabolism, transporters, transcription regulation, cell motility, and chemotaxis (Table 4). Though there were some differences in the fold changes of several significantly regulated genes between real-time PCR and RNA-Seq, the general trends were consistent between each other, suggesting that the RNA-Seq data were convincible (Additional file 11: Table S5). These differences were probably caused by use of different methods, which could be also observed in previous studies [9, 38].

**Table 3:** Numbers of significantly differentially expressed genes in the presence and absence of root exudates

Items	Up-regulated	Down-regulated
RE/Control_24h	382 (9.4 %)	261 (6.4 %)
RE/Control_48h	260 (6.4 %)	764 (18.7 %)

The percentages in parentheses represent the ratios of differentially expressed gene numbers to those of the whole genome (4,078 coding sequences)

**Table 4:** Functional categories of SQR9 genes that were significantly regulated by the maize root exudates

Functional class	24-h	48-h
1 Cell envelope and cellular processes		
1.1 Cell wall	14	39
1.2 Transport/binding proteins and lipoproteins	80	75
1.3 Sensors (signal transduction)	4	14
1.4 Membrane bioenergetics (electron transport chain and ATP synthase)	13	23
1.5 Mobility and chemotaxis	18	49
1.6 Protein secretion	1	9
1.7 Cell division	2	19
1.8 Sporulation	47	44
1.9 Germination	6	2
1.10 Transformation/competence		1
2 Intermediary metabolism		
2.1 Metabolism of carbohydrates and related molecules		
2.1.1 Specific pathway	52	41
2.1.2 Main glycolytic pathways	2	7
2.1.3 TCA cycle	1	8
2.2 Metabolism of amino acids and related molecules	43	41
2.3 Metabolism of nucleotides and nucleic acids	17	30
2.4 Metabolism of lipids	20	28
2.5 Metabolism of coenzymes and prosthetic groups	24	51
2.6 Metabolism of phosphate	2	1
2.7 Metabolism of sulfur	2	2
3 Information pathways		
3.1 DNA replication	1	12
3.2 DNA restriction/modification and repair	2	19
3.3 DNA recombination		8
3.4 DNA packaging and segregation		4
3.5 RNA synthesis	25	66
3.6 RNA modification		18
3.7 Protein synthesis	2	51
3.8 Protein modification	2	15
3.9 Protein folding	2	1
4 Other functions		
4.1 Adaptation to atypical conditions	18	25
4.2 Detoxification	18	25
4.3 Antibiotic production	6	10
4.4 Phage-related functions	15	15
4.6 Miscellaneous	4	5
Total (with known function)	443	758
5 Proteins of unknown function that are similar to other proteins		
5.1 From *Bacillus*	119	154
5.2 From other organisms	32	53
6 No similarity	49	59
Total	643	1024

Functional categories were according to SubtiList (http://genolist.pasteur.fr/SubtiList/help/function-codes.html)

### Genome and transcriptional analyses indicate that maize root exudates enhances SQR9 biofilm formation by both growth promotion and extracellular matrix induction

Bacterial biofilms are ubiquitous communities of tightly associated cells encased in an extracellular matrix [39]. Biofilm formation might be affected by both the cell population and extracellular matrix production within the communities. Here, whole transcriptomic information revealed the regulatory roles of maize root exudates on biofilm formation of SQR9.

#### Root exudates stimulates the metabolism of SQR9 in the exponential phase

**Fig. 5:**
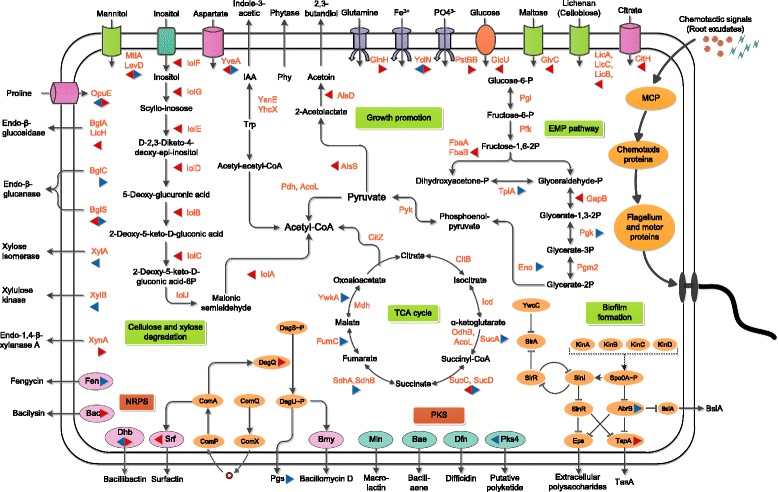
Schematic of genes involved in metabolism (EMP pathway, TCA cycle, and inositol metabolism), rhizosphere adaptation, biocontrol, and plant growth promotion in SQR9 and their expression patterns in response to root exudates. Each significantly differentially expressed gene is marked with a triangle, for which the left and right directions represent 24 h and 48 h post-inoculation, respectively. Red indicates up-regulation and blue down-regulation

**Fig. 6:**
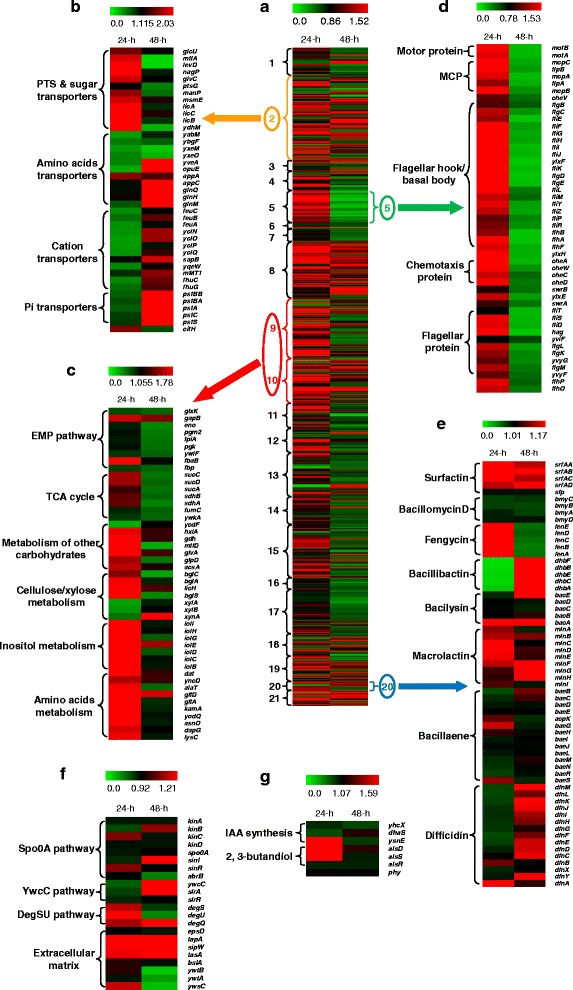
Gene expression profiles for all significant genes **a** and several representative categories. **b** Transporters. **c** Metabolism. **d** Cell motility and chemotaxis. **e** NRPS&PKS. **f** Biofilm-formation related. **g** Plant growth-promotion related. For **e-g**, all important genes (both significantly and insignificantly differentially expressed) are included. The color bar in the heatmap figures indicates the ratio of expression level of each gene between the presence of root exudates and its absence. Each number represents the category of the significantly affected genes: 1, cell wall; 2, transporters; 3, sensors; 4, membrane bioenergetics; 5, motility and chemotaxis; 6, protein secretion; 7, cell division; 8, sporulation and germination; 9, metabolism of carbohydrates and related molecules; 10, metabolism of amino acids and related molecules; 11, metabolism of nucleotides and nucleic acids; 12, metabolism of lipids; 13, metabolism of coenzymes and prosthetic groups, phosphate, and sulfur; 14, DNA replication, restriction/modification, repair, recombination, packaging and segregation; 15, RNA synthesis; 16, RNA modification; 17, protein synthesis, modification and folding; 18, adaptation to atypical conditions; 19, detoxification; 20, antibiotic production; and 21, phage-related functions

After 24 h of incubation in maize root exudate, 98 genes relevant to carbohydrates/amino acids metabolism were significantly differentially regulated; of these, 75 were activated. In detail, three genes encoding enzymes involved in the Embden-Meyerhof-Parnas (EMP) pathway (*gapB* and *fbaB*) and the tricarboxylic acid (TCA) cycle (*sucC*) were up-regulated (Figs. 5 and 6c; Additional file 12: Table S6). Other up-regulated genes related to carbohydrate metabolism included those involved in use of inositol (*iol* cluster), mannitol (*mtlD*), hexulose (*hxlA*), and other carbon sources. Also genes involved in the metabolism of amino acids, including alanine (*dat*, *alaT*), glutamate (*gltD*, *gltA*), lysine (*kamA*), and aspartate (*dapG*), were induced by root exudates (Figs. 5 and 6c; Additional file 12: Table S6). In addition, numerous genes annotated as phosphotransferase system (PTS) or sugar transporters, as well as *citH* encoding for citrate transporter, were activated by root exudates (Figs. 5 and 6b; Additional file 12: Table S6). These data, together with the observation that adding maize root exudates significantly enhanced the growth of SQR9 cells under aeration (data not shown), indicate that root exudates can stimulate the metabolism and cell growth of SQR9, thereby leading to a higher cell population.

In contrast, most of the differentially regulated genes involved in metabolism of carbohydrates (53 out of 56 genes), including seven in the EMP pathway and eight in the TCA-cycle, and amino acid/related molecules (27 of 41), were inhibited by root exudates at 48 h post-treatment (Figs. 5 and 6c; Additional file 12: Table S6). As expected, down-regulation of numerous genes related to PTS or sugar transporters was observed. In contrast, several genes involved in amino acid/peptide (*glnQHM*, *yveA*, *appC*, etc.), ferrichrome (*yclN*, *yclO*), Mg^2+^ (*sapB*), and phosphate (*pst* cluster) uptake were activated (Figs. 5 and 6b; Additional file 12: Table S6).

#### Root exudates induces extracellular matrix production by SQR9 in the stationary phase

The genetic regulation pathways of biofilm formation of *B. subtilis* (including *B. amyloliquefaciens*) have recently been summarized [39]. The SQR9 genome contains the complete set of relevant genes, including extracellular matrix production genes (*epsA-O* for exopolysaccharide synthesis, *tapA-sipW-tasA* for extracellular protein production, and *bslA* for self-assembling the bacterial hydrophobin that coats the biofilm) and regulatory genes (Spo0A ~ P-AbrB/SinI-SinR pathway, YwcC-SlrA-SlrR pathway, and DegQ pathway) (Fig. 5; Additional file 13: Table S7).

At 24 h post-inoculation, the expressions of the genes related to biofilm formation and involved in extracellular matrix production were not significantly altered, whereas at 48 h post-inoculation the *abrB* gene, a negative regulator of extracellular matrix production and root colonization [31, 39], was down-regulated by root exudates. As a result, the *tapA-sipW-tasA* operon responsible for extracellular matrix production was activated in the presence of root exudates (Figs. 5 and 6f; Additional file 12: Table S6). In addition, a small regulatory protein (*degQ*) that stimulates phosphotransfer from DegS ~ P to DegU [40], was activated. Increasing the phosphorylation level of DegU enhanced the biofilm formation and root colonization of SQR9 [37]. In summary, the RNA-Seq data suggests that maize root exudates stimulates the metabolism and growth of SQR9 in the exponential phase, whereas it induces extracellular matrix production in the stationary phrase.

### Specific components in maize root exudates stimulate biofilm formation of SQR9 through different mechanisms

For a better understanding of the chemical composition of and the presence of potential signal compounds in maize root exudates, the exudates was collected as described in the Methods and analyzed by gas chromatography–mass spectrometry (GC-MS) as described by Badri et al. [41]. The results indicated that the maize root exudates was mainly composed of carbohydrates, sugar alcohols, glycosylamines, carboxylic acids, phenolic acids, and amino acids. Glucose and xylose were found to be the most prominent carbohydrates. Also detected were amino acids (e.g., alanine, glycine), carboxylic acids (e.g., citric acid, malic acid, fumaric acid), glycerol, inositol, ethanolamine, and some other components (Additional file 14: Table S8).

**Fig. 7:**
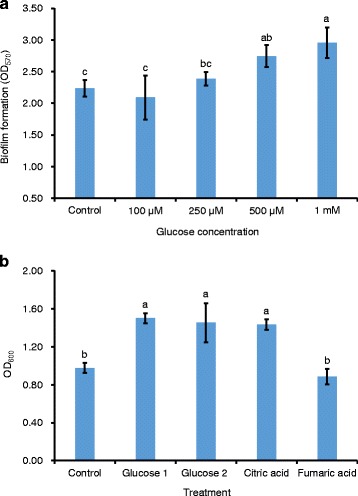
Effects of specific components in maize root exudates on biofilm formation and growth of SQR9. **a** Effects of different concentrations of glucose on the biomass of biofilm formed by SQR9 after incubation for 24 h. **b** Effects of glucose (Glucose 1, 500 μM; Glucose 2, 1 mM), citric acid (50 μM) and fumaric acid (50 μM) on the growth of SQR9 under aeration at 8 h post-inoculation. Columns with different letters are statistically different according to the Duncan’s multiple range tests (*P* < 0.05)

To further understand which maize root exudates components contributed to the SQR9 biofilm enhancement, several compounds in concentrations of 0.1–1 mM were selected for investigation based on the chemical analysis of maize root exudates. These root exudates components included glucose and xylose (the most abundant carbohydrates in the exudates); alanine, glycine, leucine, isoleucine, gamma-amino butyric acid, and valine (the most dominant amino acids); and citric acid, malic acid, and fumaric acid (important organic acids reported to be involved in plant-microbe interactions) (Additional file 14: Table S8). Only glucose at 500 μM and 1 mM significantly promoted SQR9 biofilm formation at 24 h post-inoculation, resulting in a biomass increase by 23–32 % relative to the control (Fig. 7a). No other compounds revealed significant effects, nor did glucose at 48 h post-inoculation (data not shown). Our previous study also suggested that low concentrations (~ 50 μM) of citric acid and fumaric acid enhanced the biofilm formation of SQR9 [16].

To further investigate the mechanisms by which these compounds enhanced biofilm formation, the influences of glucose, citric acid, and fumaric acid on the growth of SQR9 were assessed under aeration. Glucose (500 μM and 1 mM) and citric acid, but not fumaric acid, could significantly stimulate SQR9 cell growth (Fig. 7b).

In addition, the transcriptional levels of *epsD* and *tapA*, which are responsible for extracellular matrix production during biofilm formation, in response to glucose, citric acid, and fumaric acid, were determined by real-time PCR. Results indicated that fumaric acid induced the expression of both genes after incubation for 24 h, and citric acid stimulated *tapA* expression at 48 h post inoculation, by more than 2-fold; whereas glucose repressed the expression of *tapA* at 24 h and did not significantly affect the two operons at 48 h post-inoculation (Table 5). These findings suggested that glucose enhances the biofilm formation by growth promotion and fumaric acid stimulates biofilm formation by inducing the expressions of *epsD* and *tapA*, genes which are involved in matrix production. Citric acid seemed to use both mechanisms.

**Table 5:** Effects of glucose, citric acid, and fumaric acid on the expressions of matrix production relevant genes (*epsD* and *tapA*) of SQR9 under biofilm formation conditions

Incubation time	Treatment	Fold change in expression	
		*epsD*	*tapA*
24-h	Glucose (500 μM)	-1.82 ± 0.22	-3.62 ± 0.63*
	Citric acid (50 μM)	-1.20 ± 0.14	1.03 ± 0.08
	Fumaric acid (50 μM)	2.97 ± 0.55*	5.15 ± 0.42*
48-h	Glucose (500 μM)	-1.13 ± 0.13	-1.18 ± 0.10
	Citric acid (50 μM)	1.48 ± 0.21	2.27 ± 0.17*
	Fumaric acid (50 μM)	1.58 ± 0.30	1.31 ± 0.10

The fold changes revealed by real-time PCR of the selected genes were determined based on the threshold cycle (Ct) values and 2^-△△Ct^ method (94). Three replicates were performed for each gene. The asterisks represent a gene expression levels with fold changes > 2

### Whole transcriptomic analyses reveals the rhizosphere adaptation and plant-beneficial effects of SQR9

#### Genes involved in degradation of plant polysaccharides

Cellulose and hemicellulose are major components of root debris and are widespread in the rhizosphere [42]. Several genes involved in cellulose degradation (*bglC*, *bglS*, *bglA*, *licH*, etc.) as well as genes related to xylan transport and utilization (*xynA*, *xynB*, *xylR*, and *xylAB*), were all identified in the SQR9 genome. They are likely to improve the ability of SQR9 to exploit various plant-derived polysaccharides in the rhizosphere (Fig. 5; Additional file 13: Table S7). Interestingly, *bglS* and *bglA* were significantly induced by root exudates at 24 h post-inoculation. The same was the case for *xynA* at 48 h post-inoculation (Figs. 5 and 6c; Additional file 12: Table S6).

#### Genes involved in cell motility and chemotaxis

Bacteria in the rhizosphere sense signals released from roots [43] and swim to the root surface for attachment, which is a prerequisite for biofilm formation and root colonization [9]. In the SQR9 genome, a variety of genes governing flagellar synthesis, chemotaxis and cell motility (e.g., *fla-che* operon, *motAB*, *mcp*, *hag*, *swrA*, and *sfp*), were identified and found to be highly conserved across *Bacillus* strains (Additional file 13: Table S7) [44].

At 24 h post-inoculation, several genes involved in chemotaxis (*cheA*, *cheB*, *cheW*, *mcpB*, and *mcpC*) and flagella synthesis (*fliF-L*, *flgD*, *flgG*, *flhA*, *flhF*, and *hag*) were found to be up-regulated in response to root exudates (Fig. 6d; Additional file 12: Table S6 and Additional file 15: Figure S7), suggesting that the presence of specific compounds in maize root exudates can attract SQR9 cells. At 48 h post-inoculation, 25 of the 31 genes in the *fla-che* cluster and several other cell motility/chemotaxis genes were all down-regulated in response to the root exudates (Fig. 6d; Additional file 12: Table S6 and Additional file 15: Figure S7).

#### NRPS (nonribosomal peptide synthetase) and PKS (polyketide synthetase) gene clusters for suppressing soil-borne pathogens in the rhizosphere

A considerable proportion of the genomes of *B. amyloliquefaciens* strains are dedicated to the nonribosomal synthesis of lipopeptides and polyketides, which play important roles in suppressing of different soil-borne pathogens [5, 45]. SQR9 possesses eight gene clusters that are also present in the model PGPR *B. amyloliquefaciens* strain FZB42 [5, 45] and that are responsible for the synthesis of surfactin, bacillomycin D, fengycin, bacillibactin (siderophore), bacilysin, macrolactin, difficidin, and bacillaene, respectively (Fig. 5; Additional file 13: Table S7 and Additional file 16: Table S9). The presence of all of the antibiotic products of these clusters in SQR9 has been confirmed by high performance liquid chromatography and mass spectrometry (data not shown), and can be directly linked to the biological control activity of SQR9. Our previous results showed that bacillomycin D is the major antibiotic against the soil-borne wilt fungal pathogen *F. oxysporum* [32].

The unique genomic island GI3 (also designated as *pks4* cluster because it likely encodes the fourth polyketide antibiotic of SQR9) is composed of 30 open reading frames (ORFs) (V529_06400-06690, Additional file 17: Figure S8). BLASTP indicated that this cluster includes the genes encoding polyketide synthase modules and related proteins, as well as ABC transporters and histidine kinase, which might have been obtained from *Ornithinibacillus scapharcae* by horizontal gene transfer (Additional file 18: Table S10). An SQR9 mutant with a deletion of the whole GI3 lost its antagonistic ability against the phylogenetically closely related strain *B. amyloliquefaciens* FZB42, but not against *B. subtilis* 168, implying that this cluster might be involved in the biosynthesis of a novel polyketide antibiotic that inhibits closely-related *Bacillus* strains (data not shown). In summary, SQR9 uses approximately 9.9 % of its genome to encode a variety of antibiotics.

At 24 h post-inoculation, up-regulation of the surfactin genes *srfAA* and *srfAB* in response to the root exudates was observed. The *dhb* cluster responsible for synthesis of bacillibactin, which is a type of siderophore that operates under iron-limited conditions [5], was found to be down-regulated in the presence of root exudates. Interestingly, several genes involved in the *pks4* clusters were also down-regulated by root exudates (Figs. 5 and 6e; Additional file 12: Table S6). At 48 h post-inoculation, *dhbC*, *dhbE* were up-regulated, as well as *bacA*, which is involved in the biosynthesis of bacilysin, a dipeptide with antibacterial activity [45]. The *fen* cluster, which is responsible for the biosynthesis of fengycin, a lipodecapeptide antibiotic with an internal lactone ring and a β-hydroxy fatty acid chain that is mainly active against fungi [46], was down-regulated by the presence of the root exudates (Figs. 5 and 6e; Additional file 12: Table S6).

#### Genes involved in plant growth promotion

Several genes reported to be involved in tryptophan-dependent indole-3-acetic acid (IAA) synthesis in *B. amyloliquefaciens* FZB42, including *ysnE*, *yhcX*, and *dhaS* [5, 47], as well as the *alsRSD* operon which is responsible for 2, 3-butanediol biosynthesis, were found to be present the SQR9 genome (Fig. 5; Additional file 13: Table S7). In the transcriptomic analysis, both *alsS* and *alsD* were activated at 24 h post-inoculation, which could be attributed to presence of the precursor (tryptophane) for IAA synthesis in the maize root exudates (Figs. 5 and 6g; Additional file 12: Table S6; unpublished data).

The *phy* gene, which encodes the phytase precursor, was also detected in the SQR9 genome (Fig. 5; Additional file 13: Table S7). Phytase degrades phytate into lower phosphate esters of myo-inositol and phosphate, thus promoting plant growth under phosphate-limited condition [47].

## Discussion

Previous high-throughput studies of plant-microbe interactions under aerobic conditions *in vivo* or in the rhizosphere [8, 9, 17, 18] have indicated that biofilm formation is closely related to root colonization and is necessary for beneficial effects [34]. Although *B. amyloliquefaciens* SQR9 is a PGPR derived from the cucumber rhizosphere, it appears to have outstanding growth promotion and enhanced root colonization abilities on maize roots (Fig. 1). Considering that SQR9-derived agents are widely applied in maize production, and biofilm formation of SQR9 was significantly stimulated by maize root exudates (Fig. 2), an Illumina RNA-Seq was performed for the whole transcriptional investigation.

### Phylogenetic analysis of the *B. amyloliquefaciens* SQR9 genome

Whole-genome sequencing of SQR9 indicated that its core genome is very similar to the core genomes of other *B. amyloliquefaciens* strains, such as FZB42 and CAU B946 [5, 6]. Based on gene phylogenies, including for *gyrA* (encoding the DNA gyrase subunit A) and *cheA* (encoding the two-component sensor histidine kinase CheA), and on plant-associated characteristics, such as root colonization, nonribosomal synthesis of secondary metabolites, and the occurrence of polysaccharide-degrading enzymes, *B. amyloliquefaciens* strains can be divided into two subspecies clades, the *amyloliquefaciens* and *plantarum* groups [4]. These groups might have evolved in different environments long ago, since the patterns of molecular clock mutation and gain/loss of functional genes were quite consistent. Phylogenies of *gyrA* and *cheA* indicated that SQR9 belongs to the *plantarum* group, which consists of several plant-associated *B. amyloliquefaciens* strains, including FZB42, CAU B946, and YAU B9601-Y2 (Additional file 19: Figure S9). Several other distinguishing characteristics of these two subgroups mentioned above all supports the notion that SQR9 is more closely related to the plant-associated *B. amyloliquefaciens* strains, which are clearly distinct from the non-plant-associated strains, such as DSM7^T^, S23, and ATCC15841 [45]. Further investigation revealed that the functional elements contributing to the potential of SQR9 as a plant-associated beneficial bacterium were mainly due to genes involved in rhizosphere adaptation, biocontrol, and plant growth promotion.

### Mechanisms involved in stimulating biofilm formation by maize root exudates as revealed by RNA-Seq

Activation of the metabolism-related genes in SQR9 by root exudates at 24 h post-inoculation is consistent with previous findings that monosaccharides, amino acids, and organic acids are major components of plant root exudates and serve as growth substrates for rhizosphere microbes [9, 12, 17, 48]. Another significant group at this time-point includes genes involved in cell motility and chemotaxis, suggesting that some specific components of maize root exudates can be recognized by SQR9 to establish rhizosphere cross talking [49]. This observation is consistent with other reports showing that expression of motility-related genes is required for progression of pellicle formation [50]. Because the expressions of genes related to biofilm formation did not differ significantly from the control, the biofilm induced by root exudates at 24 h post-inoculation could be attributed to the activation of genes involved in metabolism (leading to a growth promotion and a larger cell population) and cell motility/chemotaxis. This assumption is supported by the finding that additional glucose and citric acid promoted both biofilm formation and growth of SQR9 (Fig. 7), which is also in accordance with reports in which it is reported that glucose and other carbon source can influence biofilm development in Gram-positive bacteria [51, 52]. However, glucose suppressed the expressions of *epsD* and *tapA*, genes responsible for the matrix production at 24 h post-inoculation, which could counteract the induction of these two operons by fumaric acid (Table 5).

The stimulation of biofilm formation by root exudates at 48 h post-inoculation may be mainly attributed to the suppression of *abrB*, a negative regulator of biofilm formation, which activates the *tapA-sipW-tasA* operon encoding the TasA extracellular matrix protein [39, 53]. Additional real-time PCR results also suggest that the citric acid and fumaric acid in maize root exudates enhances the expression of the *tapA* operon. Other probable reasons for this notion are: (i) repression of genes involved in cell motility and chemotaxis at 48 h, which could help to maintain the mature aggregation phase [54, 55]; (ii) down-regulation of genes related to the metabolism of carbohydrates and/or amino acids at 48-h represses microbial metabolism, which has been found to be depressed in mature biofilms relative to that of proliferating biofilms [56]; and (iii) activation of a series of genes involved in iron transport, including *dhbE*/*C* (encoding the siderophore biosynthesis protein) and *yclN*/*O* (encoding the ferrichrome ABC transporter). Iron has been reported to play important roles in bacterial biofilm formation, although its detailed function in the pellicle formation of *B. subtilis* strains has not been well explored to date [57–59]. The observation that deletion of iron transporter genes (*feuBC*, *ycgT*) in SQR9 disabled its ability to form biofilms (unpublished data), further confirms the importance of iron in SQR9 biofilm formation.

In summary, maize root exudates mediates the biofilm formation of SQR9 by both promoting cell growth and inducing matrix production. Components, including glucose and citric acid, that are directly involved in the EMP pathway and the TCA cycle stimulate cell growth during the early biofilm formation stage, whereas in the stationary stage, citric acid and other unidentified compounds activate the matrix production genes.

Although the transcriptomic data generally explain the stimulatory effects of root exudates on biofilm formation of SQR9, the detailed pathways between the signal molecules in the exudates and the target genes (e.g., *abrB*, *eps*, and *tapA* operon) remain unclear. L-Malic acid in tomato root exudates could be sensed by the extracellular calcium channels and chemotaxis receptors domain in the KinD of *B. subtilis* 3610 and could consequently stimulate the phosphorylation of Spo0A and pellicle formation [60]. Another recent study indicated that certain plant polysaccharides can trigger *B. subtilis* biofilm formation by serving as both a signal of the kinases controlling the phosphorylation state of the master regulator Spo0A as well as a source of sugars for the synthesis of the matrix exopolysaccharide [61]. These studies provide perfect models for investigating the molecular interactions between environmental signals and bacterial biofilm formation in the rhizosphere.

### NRPS/PKS clusters identified in the SQR9 genome

The eight confirmed NRPS/PKS clusters in the SQR9 genome together with another candidate encode powerful weapons to suppress various plant pathogens [45]. In addition to their antibiotic activities, these secondary metabolites were also found to have other roles in rhizosphere adaptation and indirect pathogen suppression. As a versatile lipopeptide, surfactin could affect motility by reducing surface tension [62], stimulating biofilm formation by inducing potassium leakage and the subsequent activation of downstream genes [63], and serving as a signal to induce plant resistance [64]. Bacillomycin D produced by SQR9 is involved in the early stages of biofilm formation [32].

Noticeably, GI3 encodes a potential polyketide antibiotic that inhibits closely-related *Bacillus* strains. Domain analysis and prediction of the 30 ORFs performed by Antibiotics & Secondary Metabolite Analysis Shell (antiSMASH, http://antismash.secondarymetabolites.org/) suggested that 13 modules with PKS-related domains may be involved in synthesizing a polyketide antibiotic with a 33-membered ring lactone skeleton. However, the elucidation of the molecular structure and the detailed synthesis pathway of this antibiotic will require further exploration.

### Modeling of the rhizosphere interaction of SQR9 with plants and pathogens

**Fig. 8:**
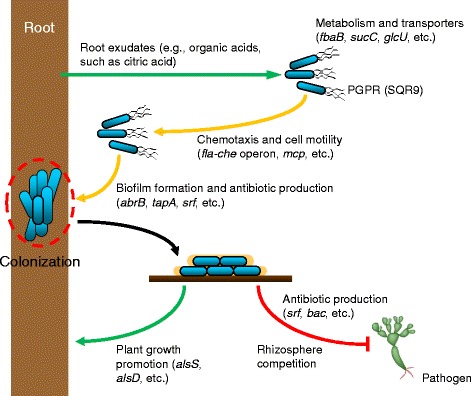
Proposed model of rhizospheric interactions of the PGPR strain, plant, and pathogens. The model is based on the results of the transcriptional profiling in this study

Whole genome sequencing and transcriptomic data of SQR9 identified several elements relevant to its potential as a plant-associated PGPR strain. Bacteria in the rhizosphere sense root exudates components released by plants [43] through methyl-accepting proteins, activate their motility related genes (e.g., *fla*-*che* operon), and then swim to the root surface for attachment. At the same time, the genes involved in metabolism (e.g., *fbaB*, *sucC*) and transport (e.g., *glcU*) of various substrates are also induced. Activation of several NRPS/PKS genes related to antibiotic production (e.g., *srf*) also takes place to outcompete other microbes in the struggle for access to the root surface and to form biofilms. Thereafter, regulation of genes related to biofilm formation in cells attached to the root surface stimulates bacterial aggregation, thus allowing effective colonization and establishing a rhizospheric competition with soil pathogens. Finally, stimulation of the NRPS/PKS and plant growth-promotion (e.g., *alsS*, *alsD*) genes contributes to pathogen biocontrol and growth stimulation, respectively (Fig. 8). Thus, root exudates can activate the rhizosphere adaptation and survival elements of SQR9, which in turn exerts beneficial biocontrol and growth-promotion effects, resulting in a mutually-beneficial relationship between plant and PGPR strain.

## Conclusion

The transcriptional profiling of *B. amyloliquefaciens* SQR9 responding to maize root exudates, and its complete genome sequence, obtained by Illumina sequencing and Roche 454 pyrosequencing, respectively, suggest that the biofilm formation-stimulation effects are mainly attributed to growth promotion and extracellular matrix induction. Future disruptions of potential rhizosphere-associated genes identified by transcriptional profiling will be performed to better understand their roles in plant-microbe interactions. In addition, an *in vivo* test will be performed in further work, and the strategy for collection of root-attached bacteria and for the elimination of plant associated cDNA reads will be carefully considered prior to any additional research.

## Methods

### Bacterial strains and culture conditions

*B. amyloliquefaciens* SQR9 (CGMCC accession No. 5808, China General Microbiology Culture Collection Center) was isolated from rhizosphere soil) [29]. SQR9 was routinely grown at 37 °C in Luria-Bertani (LB) medium, except that it was cultivated in 1/2 MSgg medium for biofilm formation experiments [2.5 mM potassium phosphate (pH 7), 50 mM MOPS (pH 7), 1 mM MgCl_2_, 350 μM CaCl_2_, 25 μM MnCl_2_, 50 μM FeCl_3_, 0.5 μM ZnCl_2_, 1 μM thiamine, 0.25 % glycerol, 0.25 % glutamate, 25 μg · mL^-1^ tryptophan, and 25 μg · mL^-1^ phenylalanine [67]. The green fluorescent protein (GFP) -labeled *B. amyloliquefaciens* SQR9 (SQR9-*gfp*) was routinely maintained in LB medium complemented with 20 μg∙mL^-1^ kanamycin.

### Plant material and growth conditions

Maize seeds (Hua’nuo Jiangnan) were surface-disinfected in 2 % NaClO solution for 10 min and rinsed four times in sterilized distilled water. Surface-sterilized seeds were pre-germinated on filter paper in a Petri dish in an incubator at 28 °C. The germinated seeds were then transferred into a box containing quartz sand and incubated in a growth chamber at 28 °C with a 16-h light/8-h dark photoperiod. After 20 day of growth, seedlings with four to five leaves were uprooted from the substrate, and their roots were gently washed to remove any adhered sands. Some of the seedlings were transplanted to pots filled with 400 g soil-less growth medium (Klasmann-Deilmann Base Substrate, Recipe-No. 422, 1:1 blended with sterile vermiculite) for growth-promotion assays. The remaining individual seedlings were transplanted into 50-mL flasks, each containing 50 mL of sterile liquid 1/2 sucrose-free Hoagland medium at 28 °C [65]. The hydroponic system was placed on a shaker (50 rpm) for 2 h each day.

### Greenhouse growth promotion assays

Roots of maize plants were dipped in one of four different suspensions: CK1 (5 mL inactivated SQR9 suspension), CK2 (10 mL inactivated SQR9), T1 (5 mL SQR9) or T2 (10 mL SQR9). Suspensions of SQR9 (10^8^ cell · mL^-1^) were prepared by shaking cells for 6 h in liquid LB medium followed by centrifugation for 10 min at 8000 × *g*. The pellet was suspended in sterile distilled water and washed twice with sterile distilled water.

Plants were irrigated regularly during the growing period. Soil in pots was fertilized with 1 % (w/w) commercial fertilizer (alkali-hydrolyzed nitrogen, 6.27 %; available phosphorus, 4.71 %; available potassium, 10.01 %). All treatments were carried out in a greenhouse at 70 % humidity under natural light at 27 ± 2 °C (day) and 22 ± 2 °C (night). Ten randomly selected plants of each treatment were harvested 55 days after transplanting, and plant height, root length and surface area, and shoot dry weight were measured. Data were analyzed using JMP software (SAS Institute, Cary, NC, USA).

### Collection of root exudates

The maize seedlings prepared above were used to collect root exudates. After incubation for 3 days, the plant roots were washed four times with sterile double-distilled water to avoid the influence of nutrient solutions. Each plant was then placed into a 50-mL flask, and the roots were submerged in 50 mL sterile double-distilled water. All plants were placed in a plant growth chamber for 24 h (16 h light/8 h dark) at 28 °C with gentle shaking at 80 rpm. The combined solutions (4000 mL from the 80 individual maize seedlings) were filtered through a 0.45 μm membrane filter (Millipore, Billerica, MA, USA), and the sterility of the exudates was judged by plating 100 μL exudates on LB medium and incubating at 30 °C for 24 h. The filter-sterilized root exudates was lyophilized and divided into two parts. One part was dissolved in sterile distilled water (50× concentrations), and the other part was kept as a powder. Both fractions were stored at -80 °C until further study.

### Colonization of maize roots by SQR9-*gfp* cells

Maize seedlings with four to five leaves, prepared as described above, were soaked in a bacterial suspension of SQR9-*gfp* (10^8^ CFU∙mL^-1^) for 30 min at 30 °C and transferred to containers with 200 g sterilized natural soil. After 5 days, the roots were collected and put on microscope slides for visualization using a confocal laser scanning microscopy (CLSM; Zeiss, Jena, Germany) to compare the thickness and the architecture of the bacterial biofilms. GFP was excited at 488 nm, and fluorescence was recorded in the range of 500–600 nm. Images were obtained using ZEN 2012 (blue edition). The density of SQR9-*gfp* on roots was also determined as described previously [66]. To this end, 0.2 g of the maize roots were homogenized in 1.8 mL of phosphate buffered saline using a mortar and pestle. The homogenates were serially diluted and plated onto LB medium containing 20 μg∙mL^-1^ kanamycin. After growth at 37 °C for 2 days, the bacterial colonies were examined using fluorescence microscopy (Olympus MVX10, Tokyo, Japan), and those emitting green fluorescence were counted.

### Effects of maize root exudates and some of its individual components on biofilm formation of SQR9

To investigate the effects of the maize root exudates on the biofilm formation of SQR9, assay was performed as described by Hamon and Lazazzera [67] in 48-well microtiter plates. An overnight culture of SQR9 was grown in LB medium at 37 °C until the OD_600_ reached 1.0. Then, the cells were centrifuged, washed twice with 1/2 MSgg, and finally resuspended in the same volume of 1/2 MSgg. Each well was filled with 1 mL 1/2 MSgg medium containing 10 μL of the suspension prepared as described above. Either 10, 20, or 40 μL of the 50× concentrated root exudates was added to the medium in the well, resulting in a final concentrations of 0.5×, 1×, and 2× root exudates relative to the concentrations in the flasks used to collect root exudates. Negative controls contained 20 μL of distilled water in each well.

After static incubation at 37 °C, the biomass of the biofilm formed by SQR9 was determined [67] after every 12-h interval until 72 h after inoculation. Growth medium and nonadherent cells were removed from the microtiter wells, which were then rinsed with distilled water. Biofilm cells were stained with 1 mL of 0.1 % crystal violet (in distilled water) for 30 min at room temperature. Excess crystal violet was poured out, and the wells were washed twice with distilled water. The bound crystal violet was solubilized with 1 mL ethanol-acetone (4:1 v/v), and the biofilm formation was quantified by measuring the OD_570_ for each well using a multi-functional plate reader Spectra Max M5 analysis system (Molecular Devices, Sunnyvale, CA, USA). Each treatment contained four biological replicates.

An additional assay was performed to evaluate the effects on SQR9 biofilm formation of several important compounds in maize root exudates, including the carbohydrates glucose and xylose; the amino acids alanine, glycine, leucine, isoleucine, γ-amino butyric acid, and valine; and the organic acids citric acid, malic acid, and fumaric acid. The final concentrations of these compounds in the wells were 100 μM, 250 μM, 500 μM, and 1 mM. Four replicates were used per treatment. After incubation for 24 and 48 h, the biofilm was quantified as described above.

Also, the influences of the compounds with biofilm-stimulation effects, including glucose (500 μM and 1 mM), citric acid (50 μM), and fumaric acid (50 μM) [16] on the growth of SQR9 were investigated under aeration (37 °C, 170 rpm). To this end, 3 mL of 1/2 MSgg medium was inoculated with a suspension of SQR9 with a final OD_600_ value of 0.01. The OD_600_ was determined at 8 h post-inoculation. Each treatment contained four replicates.

### Design of the transcriptome experiments, total RNA preparation, and microscopy

Based on the results of the pre-experiments, 1× root exudates was used as the test concentration, and the time points 24 and 48 h after incubation were selected for sampling. The biofilm formation assays were performed as described above, and 20 μL of the concentrated root exudates was added to the medium in each well, with an equal volume of distilled water in the control wells. After static incubation at 37 °C for 24 or 48 h, the SQR9 cells within the biofilms formed in the presence and absence of the root exudates were harvested, thus generating four samples. A volume of 50 mL of the culture (from the 50 wells of the microtiter plate) was mixed with 25 mL of cold “killing buffer” (20 mM Tris–HCl, 5 mM MgCl_2_, 20 mM NaN_3_, pH 7.5) and centrifuged at 4500 × *g* for 3 min at 4 °C. The pellet was then washed once more with 1 mL of “killing buffer” and immediately frozen in liquid nitrogen. The frozen cell pellets were stored at -80 °C prior to RNA isolation.

The total RNA samples were extracted using an E.Z.N.A. @ Bacterial RNA Kit (Omega, Bio-tek, Norcross, GA, USA), according to the manufacturer’s protocol. The isolated RNA was digested with DNaseI (Ambion, Carlsbad, CA, USA) to remove possible traces of DNA. The concentration of the total RNA was determined with a spectrophotometer, and its quality was checked on a 1 % agarose gel. Multiple 30-μg quantities of each RNA sample were depleted of rRNA using a Ribo-Zero™ rRNA Removal kit (Epicenter, USA) according to the manufacturer’s instructions. The resulting RNA samples were dissolved in 100 μL RNase-free water and quantified with a NanoDrop 2000 spectrophotometer (Wilmington, DE, USA).

The biofilm at 24 h post-inoculation from experiments with or without root exudates was also carefully collected and put on microscope slides for visualization with CLSM to compare the thickness and architecture, as described above.

### cDNA synthesis, construction of cDNA library, and DNA sequencing

Ten micrograms of the thermal-fragmented rRNA-depleted mRNA was incubated with biotinylated random hexamers (Illumina, San Diego, CA, USA) with the use of 1,000 units of Superscript II reverse transcriptase (Invitrogen, Carlsbad, CA, USA) for first-strand cDNA synthesis. Dynal M280 streptavidin Dynabeads (Invitrogen) were used to select the biotinylated RNA/cDNA. The first-strand of cDNA was released via alkaline hydrolysis. Subsequently, adaptors were ligated to the 5’-end of the first strand cDNA by DNA ligase (TaKaRa, Otsu, Japan), and the second-strand cDNA was synthesized through primer extension using ExTaq polymerase (TaKaRa, Japan). The synthesized cDNA was fractioned ultrasonically into 300–800 bp and purified with Ampure beads (Agencourt, USA). The prepared cDNAs were transformed into libraries using the Truseq™ DNA Sample Prep Kit-Set A (Illumina) and then clonally amplified with the TruSeq PE Cluster Kit (Illumina). DNA sequencing was performed on a HiSeq 2500 sequencing system (Illumina) by the Chinese National Human Genome Center (Shanghai, China).

### Genome sequencing and assembly

To provide the reference mapping background for the transcriptomic analysis, the genome of *B. amyloliquefaciens* SQR9 was shotgun sequenced using a Roche 454 GS FLX system (Penzberg, Germany) at the Chinese National Human Genome Center. In total, 279,622 reads produced 115.5 Mb of sequence data (28.1× coverage) with an average read length of 413 bp. The reads were assembled into 51 contigs with a total size of 4.07 Mb using Newbler software (v2.3) provided in the Roche 454 suite package [68]. Of the 51 contigs, 42 were more than 2 kb in length, and their N50 was 300.1 kb (that is, 50 % of all bases were contained in contigs of at least 300.1 kb). After linkage of the contigs, sequences obtained by the Sanger method were used to fill in gaps in the assembly and confirm regions of uncertainty.

### Genome annotation

Protein-coding genes were predicted using both Glimmer 3 [69] and Prodigal [70]. The two sets of gene calls were combined using Prodigal as the preferred start call for genes with the same stop codon. Pseudogenes and anomalous start/stop codons were identified by the GenePrimp pipeline [71]. Then, all of the genes were manually curated with the genome viewer Artemis [72]. The RNA-Seq data obtained as described below were also used to verify gene calling and identify novel transcripts, including non-coding RNAs, by Rockhopper [73]. The functional annotation was carried out using the BLASTP search tool, with *B. amyloliquefaciens* FZB42 and *B. subtilis* 168 as references, and GenBank’s non-redundant protein databases (nr) (parameters: E value: 1e-5, coverage > 60 %, identity > 50 %). Each gene was functionally classified into Cluster of Orthologous Genes (COG) categories using an RPS-BLAST search against the COG database with an E value of 1e-5 [74]. Domain prediction was also carried out with a RPS-BLAST search against the PFAM database with an E value of 1e-5 [75]. Genes for tRNAs were predicted with tRNAscan-SE [76] and for rRNAs with RNAmmer 1.2 Server [77]. Insertion sequences (ISs) were identified using a BLASTN scan against an IS database [78]. Horizontally transferred genomic islands (GIs) were identified with IslandViewer [79] and using SeqWord Sniffer tools [80] to combine the prediction results. Prophage regions were identified using the PHAST web server [81]. Dot plot comparison was implemented in MUMmer nucmer [82], and the global alignment of whole genome sequences was performed with M-GCAT software [83]. The circular map and the graphic representation of genome-compared orthologous genes were generated using Circos [84].

### Orthologous gene analysis

Orthologous genes present in the four *B. amyloliquefaciens* strains and *B. subtilis* 168 were identified using OrthoMCL [85]. The protein-coding genes of all the five bacteria were compared all-against-all using BLASTP with a minimum E value of 1e-05 and a cutoff of 70. Then, all homologous proteins were grouped into orthologous genes by the cluster tool MCL, with an inflation value of 1.5 [86]. Python scripts were developed to process the output group file, and the core genome and dispensable genome were extracted. Gene counts were based on orthology, and a Venn diagram was generated with VennDiagram [87].

### Phylogenetic analysis

A total of 18 genomes, that is, 12 *B. amyloliquefaciens* genomes, two *B. subtilis* genomes, and another four *Bacillus* genomes (*B. cereus*, *B. pumilus*, *B. licheniformis*, and *B. atrophaeus*), were included in the phylogenetic analysis. Python scripts were developed to filter the core genome to 1,044 conserved genes with exactly one member per genome; the lengths of each of these genes were nearly identical. MAFFT [88] was used to align the protein sequences and concatenate each gene alignment into a string for each genome. The interleaved NEXUS file was formatted using PAUP*4.0b10 [89]. Phylogenetic analyses of the core genome were performed using the maximum-parsimony method (observed p-distance, no evolutionary modeling required) implemented in PAUP*4.0b10 via a heuristic search (*n* = 1,000) with the random addition of sequences and the TBR tree-swapping algorithm. The reliability of the obtained clades was tested by 500 bootstrap replications. Bootstrap values > 75 % were considered significant.

### Mapping and processing of the RNA-Seq data

The clean reads obtained from Illumina sequencing (at least half of the bases with a quality > 5, not including N) were retained and mapped to the *B. amyloliquefaciens* SQR9 genome. Collected reads from different samples of each gene were transformed into reads per million reads values (RPM) [90]. Transcript abundance (fragments per kilobase unique exon sequence per megabase of library mapped; FPKM) was estimated with Cufflinks v 0.9.3 [91].

Genes exhibiting statistically significant expression differences between the control and treatment conditions were identified using MARS (MA-plot-based method with Random Sampling model) from the DEGseq program package using the false discovery rate (FDR) control method [92, 93]. Only genes that met the following filter conditions were regarded as significantly differently expressed between control and treatment: (i) fold-change ≥ 1.5; (ii) q-value ≤ 0.001 (FDR); and (iii) with a RPM consistently above 10 in at least one sample. The ratios of the gene expression levels between the treatment and control conditions belonging to different categories were used to generate a heatmap with MeV version 4.8.1 (MultiExperiment Viewer), according to the manufacturer’s instructions.

### Real-time PCR

Real-time PCR was performed on the original RNA extracts to confirm the transcriptional profiling data obtained from Illumina sequencing as well as to investigate the gene expression responses to the biofilm-stimulating compounds, including glucose (500 μM), citric acid (50 μM), and fumaric acid (50 μM) [16]. First-strand cDNA was obtained as described above. A volume of 1 μL of the cDNA was subjected to real-time PCR using SYBR Green PCR master mix (Applied Biosystems, Foster City, CA, USA). Oligonucleotide primers for the target genes were designed using Primer Premier 5 software (PREMIER Biosoft, Palo Alto, CA, USA), and the *recA* gene was used as an internal control. The reaction mixtures contained a final concentration of 25 μL SYBR^@^*Premix Ex Taq*™, 1 μL of each primer (10 μM), 1 μL ROX Reference Dye II (50×), 2 μL template DNA and 20 μL sterile water. The reactions were performed using an ABI 7500 system (Applied Biosystems) with the following conditions: an initial cycle at 95 °C for 30 s, followed by 40 cycles of 95 °C for 5 s, 65 °C for 34 s, and 72 °C for 15 s. Three technical replicates were carried out for each target gene. Quantification was analyzed based on the threshold cycle (Ct) values and the 2^-△△Ct^ method [94].

### Analysis of the composition of maize root exudates

The collected maize root exudates were analyzed by gas chromatography–mass spectrometry (GC-MS) at the Genome Center Core Services, University of California, Davis, CA, USA, as described by Badri et al. [41].

### Statistical analysis

Differences among treatments were determined by analysis of variance with Duncan’s multiple range tests (*P* < 0.05) and Student’s *t* test (*P* < 0.05 or *P* < 0.01) as appropriate. SPSS (IBM, Chicago, IL, version 19.0) was used for statistical analysis.

### Accession number

The genome sequence and annotated data of SQR9 are available in the NCBI database (accession No. CP006890).

## Additional files

Additional file 1: Figure S1. Effects of concentrations and incubation times of maize root exudates on biofilm formation of SQR9. (**A**) Influence of different concentrations of maize root exudates on SQR9 biofilm formation. Bars indicate the standard errors of the means from four replicates. Columns with different letters are statistically different according to the Duncan’s multiple range tests (*P* < 0.05, for 24 and 48 h post-inoculation, respectively). 1/2 RE, RE, and 2× RE represent that the 1/2 MSgg medium were supplied with 0.5×, 1×, and 2× concentrated root exudates as described in the Methods. (**B**) Dynamics of the biomass of biofilm-formed SQR9 in response to 1× maize root exudates. Bars indicate the standard errors of the means from four replicates. The arrows represent the sampling times for transcriptional profiling analysis. (DOCX 44 kb) Additional file 2: Table S1. Novel noncoding RNAs predicted by Rockhopper. The expression level listed in the table was obtained by Rockhopper. Con_24, negative control, 24 h post- inoculation; Ma_24, maize root exudates treatment, 24 h post-inoculation; Con_48, negative control, 48 h post-inoculation; Ma_48, maize root exudates treatment, 48 h post-inoculation. (XLSX 26 kb) Additional file 3: Table S2. Unique genes in *Bacillus amyloliquefaciens* SQR9 in the pan genome formed by SQR9, *Bacillus subtilis* 168, and *B. amyloliquefaciens* FZB42, DSM7^T^, and B9601-Y2. (XLSX 36 kb) Additional file 4: Figure S2. Comparison of the genomes of *Bacillus amyloliquefaciens* strains SQR9 and FZB42 and *Bacillus subtilis* 168. (**A** and **B**) Matches of the SQR9 genome with those of *B. subtilis* 168 (A) and *B. amyloliquefaciens* FZB42 (**B**). Synteny plots show the comparison at the nucleotide level of the *B. amyloliquefaciens* SQR9 genome (a and b, vertical axis) with the genomes of *B. subtilis* 168 (A, horizontal axis) and *B. amyloliquefaciens* FZB42 (B, horizontal axis). Forward matches are plotted in red and reverse matches in blue. (**C**) Global alignment of the three strains built by the M-GCAT program. (DOCX 295 kb) Additional file 5: Table S3. Genes shared by *Bacillus amyloliquefaciens* SQR9 and *B. amyloliquefaciens* FZB42 but absent in *Bacillus subtilis* 168 (Sheet 1), or shared by SQR9 and 168 but absent in FZB42 (Sheet 2). (XLSX 77 kb) Additional file 6: Figure S3. Phylogenetic tree drawn from 1,044 conserved genes of the core genomes of 18 *Bacillus* genomes. The maximum-parsimony tree was obtained in PAUP*4.0b10 via a heuristic search (*n* = 1,000) with the random addition of sequences and the TBR tree-swapping algorithm. Bootstrap values of > 75 % were considered significant. *Bacillus cereus* ATCC 14579 was used as the outgroup. (DOCX 55 kb) Additional file 7: Table S4. Genomic island (GI) prediction of *Bacillus amyloliquefaciens* SQR9. GIs were predicted by IslandViewer and M-GCAT (Sheet 1). Sheet 2 shows the detailed information for all genes in the 11 predicted GIs. (XLSX 209 kb) Additional file 8: Figure S4. Orthologous genes in *Bacillus subtilis* 168 and *Bacillus amyloliquefaciens* SQR9 and FZB42. Lines in blue represent orthologs between SQR9 and FZB42 that were not found in 168, while lines in red represent orthologs between SQR9 and 168 that were not found in FZB42. Highlight-1 is genomic island 3 of SQR9, which was only found in SQR9, and highlight-2 shows the prophage (genomic island 7, 8, and 9) only shared by SQR9 and 168. (DOCX 378 kb) Additional file 9: Figure S5. Saturation curves of the Illumina RNA-Seq data. The X-axis represents the number of reads. The Y-axis represents the number of open reading frames covered by the reads obtained. (DOCX 90 kb) Additional file 10: Figure S6. Comparison of different gene expression patterns regulated by root exudates at 24 h (**A**) and 48 h (**B**) post-inoculation. Every point represents a gene with different expression levels (fragments per kilobase unique exon sequence per megabase of library mapped; FPKM) in two transcriptomes. Blue color denotes genes with no significant differences between the two transcriptomes, red means up-regulation and green means down-regulation. (DOCX 228 kb) Additional file 11: Table S5. Comparison of fold-changes of differentially expressed genes obtained by Illumina RNA-Seq and real-time PCR. The fold changes revealed by real-time PCR of the selected genes were determined based on the threshold cycle (Ct) values and 2^-△△Ct^ method. Three replicates were performed for each gene. (DOCX 15 kb) Additional file 12: Table S6. Illumina RNA-Seq data of all significantly differentially expressed genes in different categories (Sheet 1, 24 h post-inoculation; Sheet 2, 48 h post-inoculation). (XLSX 275 kb) Additional file 13: Table S7. List of genes involved in rhizosphere adaptation (degradation of plant polysaccharides, cell motility and chemotaxis, biofilm formation, transporters, and detoxification), synthesis of secondary metabolisms, and plant growth promotion. (XLSX 59 kb) Additional file 14: Table S8. Composition of maize root exudates analyzed by gas chromatography–mass spectrometry. (XLSX 55 kb) Additional file 15: Figure S7. Diagram of genes involved in cell motility (mainly flagellar synthesis, **A** and **B**) and chemotaxis (**C** and **D)**, and their expression patterns in response to root exudates. The significantly regulated genes (red for up-regulation and green for down-regulation) were mapped in the KEGG pathway (with some modifications for the flagellar synthesis). (**A**) and (**C**) show expression patterns at 24 h post-inoculation and (**B**) and (**D**) at 48 h post-inoculation. (DOCX 114 kb) Additional file 16: Table S9. NRPS and PKS gene clusters involved in the biosynthesis of secondary metabolites in *Bacillus amyloliquefaciens* SQR9 and FZB42. (DOCX 15 kb) Additional file 17: Figure S8. Schematic representation of genes in the *pks4* cluster in *Bacillus amyloliquefaciens* SQR9. Non-ribosomal polyketide synthetase/polyketide synthetase is marked in red, transporter genes in blue, accessory genes in green, and hypothetical genes in yellow. (DOCX 27 kb) Additional file 18: Table S10. BLASTP results for each open reading frame in the *pks4* cluster (XLSX 13 kb) Additional file 19: Figure S9. Neighbor-joining phylogenetic tree based on partial *gyrA* (**A**) and *cheA* (**B**) nucleotide sequences. The consensus tree was reconstructed from 1,000 trees according to the extended majority rule (SEQBOOT program). Bootstrap values >50 % (1,000 repetitions) are indicated at branch points. (DOCX 136 kb)

## Abbreviations

ABC: ATP-binding cassette
CLSM: Confocal laser scanning microscopy
EMP: Embden-Meyerhof-Parnas
FDR: False discovery rate
FOC: *Fusarium oxysporum* f. sp. *cucumerinum* J. H. Owen
FPKM: Fragments per kilobase unique exon sequence per megabase of library mapped
GFP: Green fluorescence protein
GI: Genomic island
IAA: Indole-3-acetic acid
NGS: Next-generation sequencing
NRPS: Nonribosomal peptide synthetase
ORF: Open reading frame
PGPR: Plant growth-promoting rhizobacteria
PKS: Polyketide synthase
PTS: Phosphotransferase system
RPM: Reads per million
TCA: Tricarboxylic acid
