# Supplementary material for: Whole transcriptomic analysis of the plant-beneficial rhizobacterium Bacillus amyloliquefaciens SQR9 during enhanced biofilm formation regulated by maize root exudates
Source: BMC Genomics. 2015 Sep 7;16(1):685. doi: 10.1186/s12864-015-1825-5 (PMC4562157; doi:10.1186/s12864-015-1825-5)
Supplement: Additional file 1: Figure S1. — Effects of concentrations and incubation times of maize root exudates on biofilm formation of SQR9. (A) Influence of different concentrations of maize root exudates on SQR9 biofilm formation. Bars indicate the standard errors of the means from four replicates. Columns with different letters are statistically different according to the Duncan’s multiple range tests (P < 0.05, for 24 and 48 h post-inoculation, respectively). 1/2 RE, RE, and 2× RE represent that the 1/2 MSgg medium were supplied with 0.5×, 1×, and 2× concentrated root exudates as described in the Methods. (B) Dynamics of the biomass of biofilm-formed SQR9 in response to 1× maize root exudates. Bars indicate the standard errors of the means from four replicates. The arrows represent the sampling times for transcriptional profiling analysis. (DOCX 44 kb) [file 12864_2015_1825_MOESM1_ESM.docx]

**A**

48-h

24-h

**B**

**Figure S1 Effects of concentrations and incubation times of maize root exudates on biofilm formation of SQR9. (A)** Influence of different concentrations of maize root exudates on SQR9 biofilm formation. Bars indicate the standard errors of the means from four replicates. Columns with different letters are statistically different according to the Duncan’s multiple range tests (*P* < 0.05, for 24 and 48 h post-inoculation, respectively). 1/2 RE, RE, and 2× RE represent that the 1/2 MSgg medium were supplied with 0.5×, 1×, and 2× concentrated root exudates as described in the Methods. **(B)** Dynamics of the biomass of biofilm-formed SQR9 in response to 1× maize root exudates. Bars indicate the standard errors of the means from four replicates. The arrows represent the sampling times for transcriptional profiling analysis.
