# Supplementary material for: Whole transcriptomic analysis of the plant-beneficial rhizobacterium Bacillus amyloliquefaciens SQR9 during enhanced biofilm formation regulated by maize root exudates
Source: BMC Genomics. 2015 Sep 7;16(1):685. doi: 10.1186/s12864-015-1825-5 (PMC4562157; doi:10.1186/s12864-015-1825-5)
Supplement: Additional file 4: Figure S2. — Comparison of the genomes of Bacillus amyloliquefaciens strains SQR9 and FZB42 and Bacillus subtilis 168. (A and B) Matches of the SQR9 genome with those of B. subtilis 168 (A) and B. amyloliquefaciens FZB42 (B). Synteny plots show the comparison at the nucleotide level of the B. amyloliquefaciens SQR9 genome (a and b, vertical axis) with the genomes of B. subtilis 168 (A, horizontal axis) and B. amyloliquefaciens FZB42 (B, horizontal axis). Forward matches are plotted in red and reverse matches in blue. (C) Global alignment of the three strains built by the M-GCAT program. (DOCX 295 kb) [file 12864_2015_1825_MOESM4_ESM.docx]

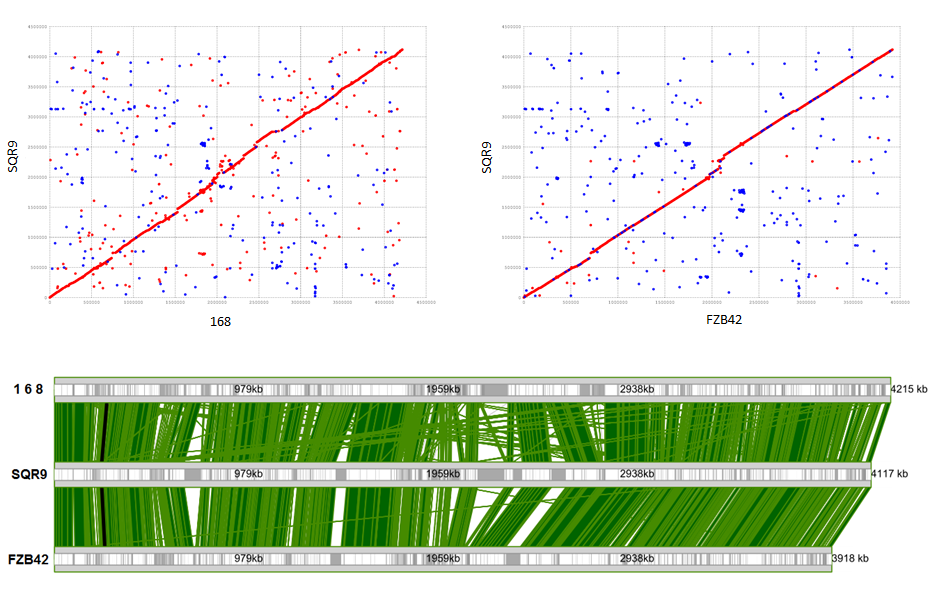


**B**

**C**

**A**

**Figure S2 Comparison of the genomes of *Bacillus amyloliquefaciens* strains SQR9 and FZB42 and *Bacillus subtilis* 168. (A and B)** Matches of the SQR9 genome with those of *B. subtilis* 168 **(A)** and *B. amyloliquefaciens* FZB42 **(B)**. Synteny plots show the comparison at the nucleotide level of the *B. amyloliquefaciens* SQR9 genome (a and b, vertical axis) with the genomes of *B. subtilis* 168 (A, horizontal axis) and *B. amyloliquefaciens* FZB42 (B, horizontal axis). Forward matches are plotted in red and reverse matches in blue. **(C)** Global alignment of the three strains built by the M-GCAT program.
