# Supplementary material for: Whole transcriptomic analysis of the plant-beneficial rhizobacterium Bacillus amyloliquefaciens SQR9 during enhanced biofilm formation regulated by maize root exudates
Source: BMC Genomics. 2015 Sep 7;16(1):685. doi: 10.1186/s12864-015-1825-5 (PMC4562157; doi:10.1186/s12864-015-1825-5)
Supplement: Additional file 6: Figure S3. — Phylogenetic tree drawn from 1,044 conserved genes of the core genomes of 18 Bacillus genomes. The maximum-parsimony tree was obtained in PAUP*4.0b10 via a heuristic search (n = 1,000) with the random addition of sequences and the TBR tree-swapping algorithm. Bootstrap values of > 75 % were considered significant. Bacillus cereus ATCC 14579 was used as the outgroup. (DOCX 55 kb) [file 12864_2015_1825_MOESM6_ESM.docx]

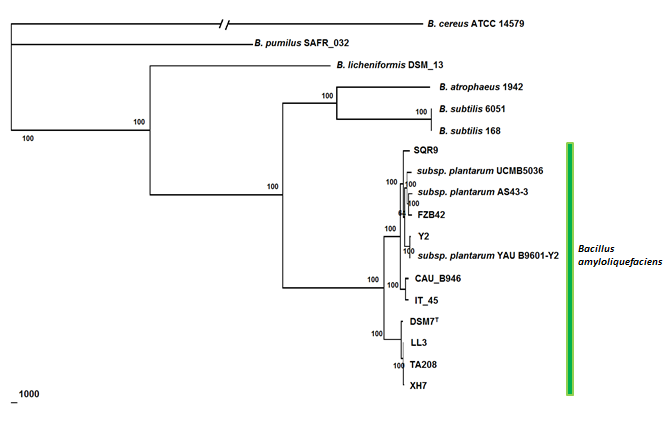


**Figure S3** **Phylogenetic tree drawn from 1,044 conserved genes of the core genomes of 18 *Bacillus* genomes.** The maximum-parsimony tree was obtained in PAUP*4.0b10 via a heuristic search (n=1,000) with the random addition of sequences and the TBR tree-swapping algorithm. Bootstrap values of >75% were considered significant. *Bacillus cereus* ATCC 14579 was used as the outgroup.
