# Supplementary material for: Whole transcriptomic analysis of the plant-beneficial rhizobacterium Bacillus amyloliquefaciens SQR9 during enhanced biofilm formation regulated by maize root exudates
Source: BMC Genomics. 2015 Sep 7;16(1):685. doi: 10.1186/s12864-015-1825-5 (PMC4562157; doi:10.1186/s12864-015-1825-5)
Supplement: Additional file 8: Figure S4. — Orthologous genes in Bacillus subtilis 168 and Bacillus amyloliquefaciens SQR9 and FZB42. Lines in blue represent orthologs between SQR9 and FZB42 that were not found in 168, while lines in red represent orthologs between SQR9 and 168 that were not found in FZB42. Highlight-1 is genomic island 3 of SQR9, which was only found in SQR9, and highlight-2 shows the prophage (genomic island 7, 8, and 9) only shared by SQR9 and 168. (DOCX 378 kb) [file 12864_2015_1825_MOESM8_ESM.docx]

**
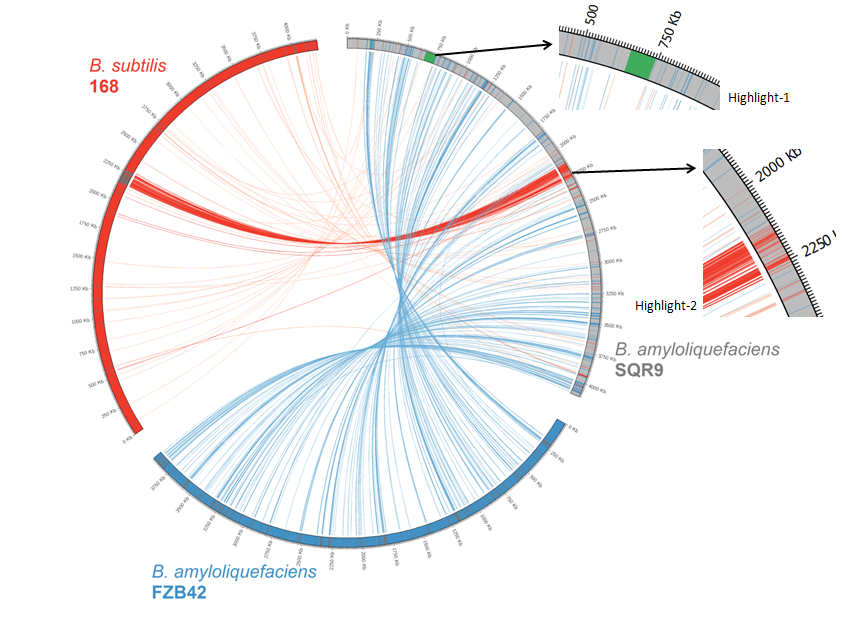
**

**Figure S4 Orthologous genes in *Bacillus subtilis* 168 and *Bacillus amyloliquefaciens* SQR9 and FZB42.** Lines in blue represent orthologs between SQR9 and FZB42 that were not found in 168, while lines in red represent orthologs between SQR9 and 168 that were not found in FZB42. Highlight-1 is genomic island 3 of SQR9, which was only found in SQR9, and highlight-2 shows the prophage (genomic island 7, 8, and 9) only shared by SQR9 and 168.
