# Supplementary material for: Whole transcriptomic analysis of the plant-beneficial rhizobacterium Bacillus amyloliquefaciens SQR9 during enhanced biofilm formation regulated by maize root exudates
Source: BMC Genomics. 2015 Sep 7;16(1):685. doi: 10.1186/s12864-015-1825-5 (PMC4562157; doi:10.1186/s12864-015-1825-5)
Supplement: Additional file 9: Figure S5. — Saturation curves of the Illumina RNA-Seq data. The X-axis represents the number of reads. The Y-axis represents the number of open reading frames covered by the reads obtained. (DOCX 90 kb) [file 12864_2015_1825_MOESM9_ESM.docx]

**
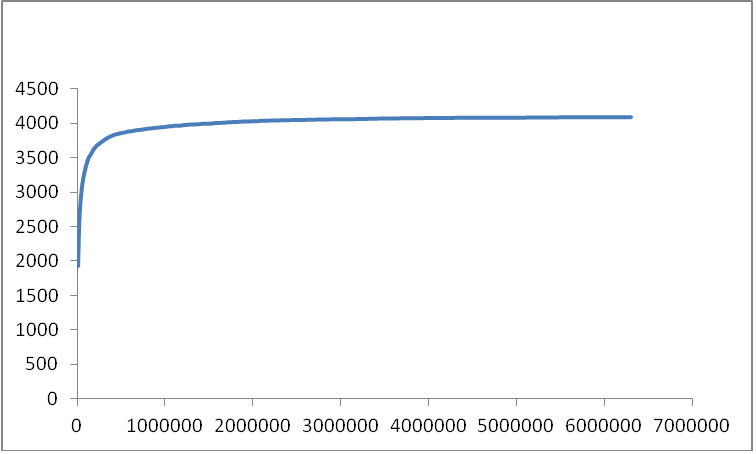

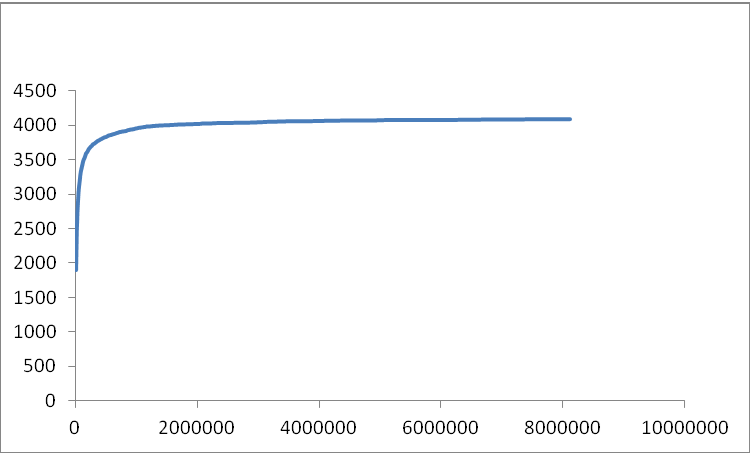
**

Maize exudates_24-h

Control_24-h

**
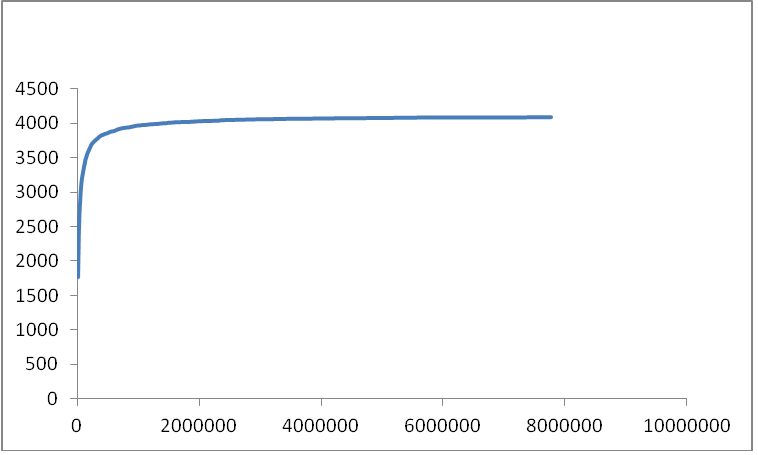

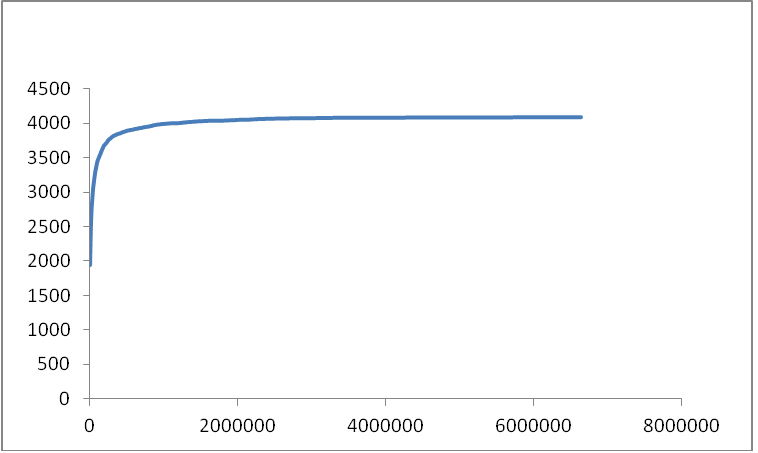
**

Maize exudates_48-h

Control_48-h

**Figure S5 Saturation curves of the Illumina RNA-Seq data.** The X-axis represents the number of reads. The Y-axis represents the number of open reading frames covered by the reads obtained.
