# Supplementary material for: Whole transcriptomic analysis of the plant-beneficial rhizobacterium Bacillus amyloliquefaciens SQR9 during enhanced biofilm formation regulated by maize root exudates
Source: BMC Genomics. 2015 Sep 7;16(1):685. doi: 10.1186/s12864-015-1825-5 (PMC4562157; doi:10.1186/s12864-015-1825-5)
Supplement: Additional file 10: Figure S6. — Comparison of different gene expression patterns regulated by root exudates at 24 h (A) and 48 h (B) post-inoculation. Every point represents a gene with different expression levels (fragments per kilobase unique exon sequence per megabase of library mapped; FPKM) in two transcriptomes. Blue color denotes genes with no significant differences between the two transcriptomes, red means up-regulation and green means down-regulation. (DOCX 228 kb) [file 12864_2015_1825_MOESM10_ESM.docx]

Down-regulation:

261 (6.4%)

Up-regulation: 382 (9.4%)

**A**

Up-regulation: 260 (6.4%)

Down-regulation:

764 (18.7%)

**B**

**Figure S6 Comparison of different gene expression patterns regulated by root exudates at 24 h (A) and 48 h (B) post-inoculation.** Every point represents a gene with different expression levels (fragments per kilobase unique exon sequence per megabase of library mapped; FPKM) in two transcriptomes. Blue color denotes genes with no significant differences between the two transcriptomes, red means up-regulation and green means down-regulation.
