# Supplementary material for: Whole transcriptomic analysis of the plant-beneficial rhizobacterium Bacillus amyloliquefaciens SQR9 during enhanced biofilm formation regulated by maize root exudates
Source: BMC Genomics. 2015 Sep 7;16(1):685. doi: 10.1186/s12864-015-1825-5 (PMC4562157; doi:10.1186/s12864-015-1825-5)
Supplement: Additional file 11: Table S5. — Comparison of fold-changes of differentially expressed genes obtained by Illumina RNA-Seq and real-time PCR. The fold changes revealed by real-time PCR of the selected genes were determined based on the threshold cycle (Ct) values and 2-△△Ct method. Three replicates were performed for each gene. (DOCX 15 kb) [file 12864_2015_1825_MOESM11_ESM.docx]

**Table S5 Comparison of fold-changes of differentially expressed genes obtained by Illumina RNA-Seq and real-time PCR.** The fold changes revealed by real-time PCR of the selected genes were determined based on the threshold cycle (Ct) values and 2^-△△­­Ct^ method. Three replicates were performed for each gene.

| **Gene** | **RNA-Seq** | **real-time PCR** |
| --- | --- | --- |
| **24-h** |  |  |
| *yusK* | 2.07 | 2.21 ± 0.53 |
| *lrgB* | 2.42 | 1.31 ± 0.14 |
| *mcpC* | 2.10 | 7.60 ± 1.63 |
| *fbaB* | 2.32 | 14.14 ± 0.83 |
| *nagP* | 2.15 | 2.69 ± 0.32 |
| *sucC* | 1.51 | 2.05 ± 0.28 |
| *citH* | 1.59 | 1.78 ± 0.14 |
| *gapB* | 1.63 | 11.03 ± 1.67 |
| *srfAA* | 1.80 | 2.82 ± 0.07 |
| *iolE* | 6.46 | 2.76 ± 0.47 |
| *bglS* | 1.92 | 3.29 ± 0.31 |
| *dhbF* | -7.89 | -16.50 ± 1.78 |
| **48-h** |  |  |
| *yusK* | -2.09 | -10.46 ± 1.71 |
| *mcpC* | -2.24 | -11.77 ± 2.91 |
| *abrB* | -2.17 | -2.85 ± 0.47 |
| *yqxM* | 1.85 | 4.32 ± 0.53 |
| *cheA* | -4.58 | -4.10 ± 0.30 |
| *cheD* | -2.45 | -4.19 ± 1.31 |
| *pstBB* | -4.60 | 5.76 ± 0.08 |
| *resE* | -2.48 | -5.14 ± 0.20 |
| *sucC* | -1.76 | -5.07 ± 1.18 |
