# Supplementary material for: Whole transcriptomic analysis of the plant-beneficial rhizobacterium Bacillus amyloliquefaciens SQR9 during enhanced biofilm formation regulated by maize root exudates
Source: BMC Genomics. 2015 Sep 7;16(1):685. doi: 10.1186/s12864-015-1825-5 (PMC4562157; doi:10.1186/s12864-015-1825-5)
Supplement: Additional file 15: Figure S7. — Diagram of genes involved in cell motility (mainly flagellar synthesis, A and B) and chemotaxis (C and D), and their expression patterns in response to root exudates. The significantly regulated genes (red for up-regulation and green for down-regulation) were mapped in the KEGG pathway (with some modifications for the flagellar synthesis). (A) and (C) show expression patterns at 24 h post-inoculation and (B) and (D) at 48 h post-inoculation. (DOCX 114 kb) [file 12864_2015_1825_MOESM15_ESM.docx]

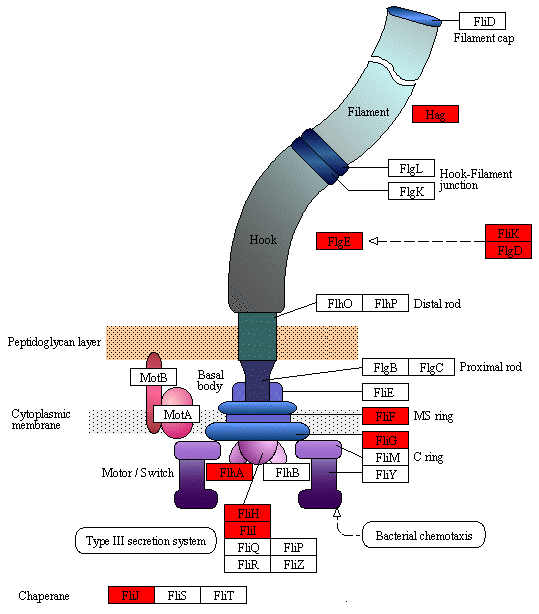

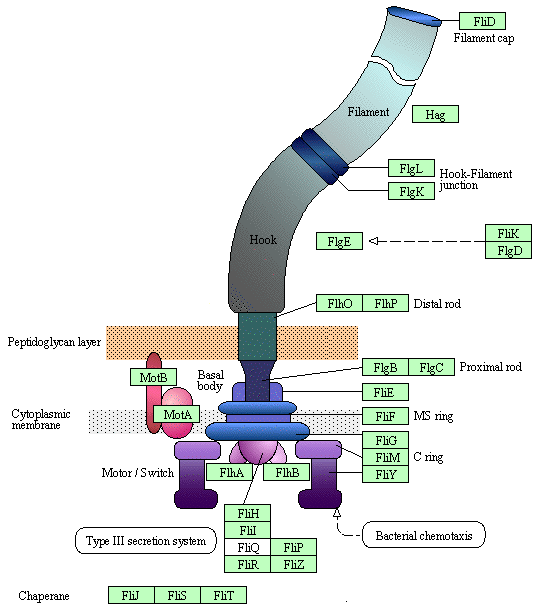


**B**

**A**


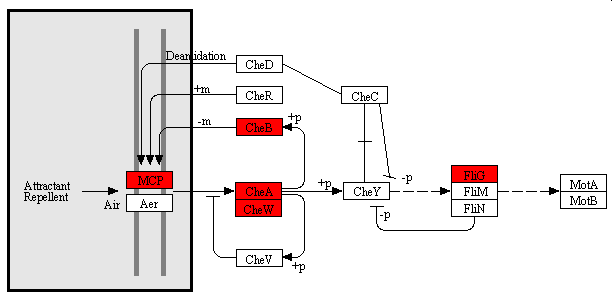


**C**


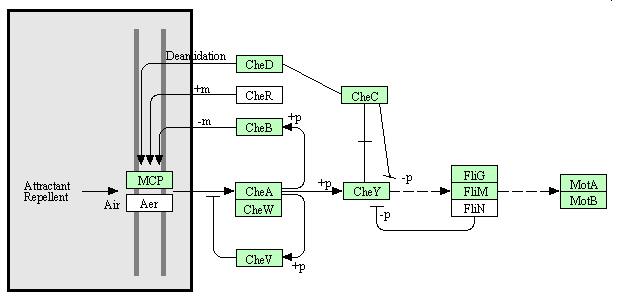


**D**

**Figure S7 Diagram of genes involved in cell motility (mainly flagellar synthesis, A and B) and chemotaxis (C and D), and their expression patterns in response to root exudates.** The significantly regulated genes (red for up-regulation and green for down-regulation) were mapped in the KEGG pathway (with some modifications for the flagellar synthesis). **(A)** and **(C)** show expression patterns at 24 h post-inoculation and **(B)** and **(D)** at 48 h post-inoculation.
