# Supplementary material for: Whole transcriptomic analysis of the plant-beneficial rhizobacterium Bacillus amyloliquefaciens SQR9 during enhanced biofilm formation regulated by maize root exudates
Source: BMC Genomics. 2015 Sep 7;16(1):685. doi: 10.1186/s12864-015-1825-5 (PMC4562157; doi:10.1186/s12864-015-1825-5)
Supplement: Additional file 16: Table S9. — NRPS and PKS gene clusters involved in the biosynthesis of secondary metabolites in Bacillus amyloliquefaciens SQR9 and FZB42. (DOCX 15 kb) [file 12864_2015_1825_MOESM16_ESM.docx]

**Table S9 NRPS and PKS gene clusters involved in the biosynthesis of secondary metabolites in *Bacillus amyloliquefaciens* SQR9 and FZB42.**

| **Compound** | **Enzymes** | **SQR9** | **Size(kb)** | **Dependent on** | **FZB42** | **Identity %** |
| --- | --- | --- | --- | --- | --- | --- |
| Surfactin | NRPS | *srfAA, AB, AC, AD, sfp* | 28.3 | Sfp | *srfAA, AB, AC, AD, sfp* | 98-99 |
| Bacillomycin D | NRPS/PKS | *bmyCBAD* | 37.7 | Sfp, YczE | *bmyCBAD* | 98-99 |
| Fengycin | NRPS | *fenABCDE* | 37.7 | Sfp | *fenABCDE* | 97-98 |
| Bacillibactin | NRPS | *dhbACEBF* | 11.7 | Sfp | *dhbACEBF* | 98-99 |
| Bacilysin | NRPS | *bacABCDE, ywfG* | 5.9 | - | *bacABCDE, ywfG* | 98-99 |
| Macrolactin | PKS | *mlnABCDEFGHI* | 53.2 | Sfp, YczE | *mlnABCDEFGHI* | 98 |
| Bacillaene | PKS/NRPS | *baeBCDE,acpK,baeGHIJLMNRS* | 72.4 | Sfp, YczE | *baeBCDE,acpK,baeGHIJLMNRS* | 98-99 |
| Difficidin | PKS | *dfnAYXBCDEFGHIJKLM* | 69.5 | Sfp, YczE | *dfnAYXBCDEFGHIJKLM* | 98-99 |
| Unknown | PKS | *ccmA, fabD, pksG, etc* | 85.8 | Unknown | Not present | 0 |
