# Supplementary material for: Whole transcriptomic analysis of the plant-beneficial rhizobacterium Bacillus amyloliquefaciens SQR9 during enhanced biofilm formation regulated by maize root exudates
Source: BMC Genomics. 2015 Sep 7;16(1):685. doi: 10.1186/s12864-015-1825-5 (PMC4562157; doi:10.1186/s12864-015-1825-5)
Supplement: Additional file 17: Figure S8. — Schematic representation of genes in the pks4 cluster in Bacillus amyloliquefaciens SQR9. Non-ribosomal polyketide synthetase/polyketide synthetase is marked in red, transporter genes in blue, accessory genes in green, and hypothetical genes in yellow. (DOCX 27 kb) [file 12864_2015_1825_MOESM17_ESM.docx]

**Figure S8 Schematic representation of genes in the *pks4* cluster in *Bacillus amyloliquefaciens* SQR9.** Non-ribosomal polyketide synthetase/polyketide synthetase is marked in red, transporter genes in blue, accessory genes in green, and hypothetical genes in yellow.
