# Supplementary material for: Whole transcriptomic analysis of the plant-beneficial rhizobacterium Bacillus amyloliquefaciens SQR9 during enhanced biofilm formation regulated by maize root exudates
Source: BMC Genomics. 2015 Sep 7;16(1):685. doi: 10.1186/s12864-015-1825-5 (PMC4562157; doi:10.1186/s12864-015-1825-5)
Supplement: Additional file 19: Figure S9. — Neighbor-joining phylogenetic tree based on partial gyrA (A) and cheA (B) nucleotide sequences. The consensus tree was reconstructed from 1,000 trees according to the extended majority rule (SEQBOOT program). Bootstrap values >50 % (1,000 repetitions) are indicated at branch points. (DOCX 136 kb) [file 12864_2015_1825_MOESM19_ESM.docx]

**A**

*B. cereus* ATCC 14579 (BC0006)

UCM B-5121 (AY212981)

UCM B-5017 (AY212970)

UCM B-5113 (AY212974)

At4 (AY212985)

At1 (AY212978)

UCM B-5033 (AY212973)

FZB24 (FN652782)

FZB13 (FN652781)

FZB42 (ABS72455)

DJ-5 (AY822026)

FZB45 (FN662840)

SQR9 (V529_00070)

UCM B-5036 (AY212975)

UCM B-5044 (AY212976)

FZB113 (FN662843)

CAU B946 (FN652789)

NAU B85 (FN662842)

FZB109 (FN662837)

NAU B2 (FN662841)

YAU B9601-Y2 (FN652790)

S23 (FN652780.2)

NRRL B14393 (EU138596)

DSM7 (CBI41133)

ATCC15841 (FN662838)

NRRL BD-601 (EU138645)

*B. vallismortis* NRRL B-14890T (AF272025)

*B. subtilis subsp. inaquosorum* BD-571 (GQ488739)

*B. subtilis subsp. inaquosorum* B-23056 (GQ488740)

*B. subtilis subsp. spizizenii* NRRL B-23049T (AF272020)

*B. subtilis* UCM B-5008 (AY212969)

*B. subtilis* At3 (AY212982)

*B. subtilis subsp. subtilis* UCM B-5184 (AY212979)

*B. subtilis subsp. subtilis* 168 (CAB11783)

*B. subtilis subsp. subtilis* At5 (AY212984)

*B. subtilis subsp. subtilis* At2 (AY212983)

*B. mojavensis* NRRL BD-600 (EU138644)

*B. mojavensis* NRRL BD-600 (EU138644)

*B. atrophaeus* NRRL NRS-213 (EU138654)

*B. atrophaeus* NRRL BD-622 (EU138651)

*B. atrophaeus* NRRL BD-622 (EU138651)

65

49

34

100

96

66

67

81

78

37

27

62

99

69

100

76

49

63

80

99

14

10

29

54

81

21

46

28

46

45

49

31

90

22

22

0.05

**B**

*B. cereus* ATCC 14579^T^ (BC_1628)

DSM7^T^ (CBI42841)

S23 (FN652791)

ATCC15841 (FN652792)

FZB109 (FN652795)

YAU B9601-Y2 (FN652804)

NAU B2 (FN652799)

NAU B3 (FN652800)

CAU B946 (FN652803)

NAU B55 (FN652801)

FZB111 (FN652796)

UCM B-5033 (AY212957)

UCM B-5113 (AY212962)

UCM B-5017 (AY212954)

UCM B-5044 (AY212956)

UCM B-5036 (AY212953)

SQR9 (V529_15840)

FZB113 (FN652797)

FZB13 (FN652793)

FZB42^T^ (ABS73990)

FZB24 (FN652794)

*B. mojavensis* UCM B-5051 (AY212959)

*B. mojavensis* DSMZ 9205 (AY212965)

*B. mojavensis* UCM B-5075 (AY212963)

*B. subtilis subsp. subtilis* 168 (CAB13516.2)

*B. subtilis subsp. subtilis* UCM B-5049 (AY212958)

*B. subtilis subsp. subtilis* UCM B-5137 (AY212960)

*B. subtilis subsp. spizizenii* DSMZ 397 (AY212966)

*B. subtilis subsp. spizizenii* UCM B-5014 (AY212955)

*B. subtilis* UCM B-5008 (AY212952)

*B. subtilis subsp. spizi*zenii ACF (DQ219359)

78

100

100

74

75

100

99

100

61

86

100

100

93

81

90

75

85

97

98

57

89

64

62

82

79

83

0.1

**Figure S9 Neighbor-joining phylogenetic tree based on partial *gyrA* (A) and *cheA* (B) nucleotide sequences.** The consensus tree was reconstructed from 1,000 trees according to the extended majority rule (SEQBOOT program). Bootstrap values >50% (1,000 repetitions) are indicated at branch points.
